# Gold Polymer Nanomaterials: A Promising Approach for Enhanced Biomolecular Imaging

**DOI:** 10.7150/ntno.89087

**Published:** 2024-01-01

**Authors:** Panangattukara Prabhakaran Praveen Kumar, Ritu Mahajan

**Affiliations:** 1 KU-KIST Graduate School of Converging Science and Technology, Korea University, 145, Anam-ro, Seongbuk-gu, Seoul 02841, Korea.; 2 Technology Business Incubator (TBI), Indian Institute of Science Education and Research (IISER), Mohali Knowledge City, Sector 81, SAS Nagar, Mohali, Manauli PO 140306, Punjab India.

## Abstract

Gold nanoparticles (AuNPs) possess unique optical properties, making them highly attractive nanomaterials for biomedical research. By exploiting the diverse optical characteristics of various gold nanostructures, significant enhancements can be achieved in biosensing and biomedical imaging fields. The potential of AuNPs can be enhanced by creating hybrid nanocomposites with polymers, which offer supplementary functionalities, responsiveness, and enhanced biocompatibility. Moreover, polymers can modify the surface charges of AuNPs, thereby improving or controlling the efficiency of cellular uptake and the distribution of these nanoparticles within the body. Polymer modification using AuNPs offers a wide array of benefits, including improved sensitivity, specificity, speed, contrast, resolution, and penetration depth. By incorporating AuNPs into the polymer matrix, these enhancements synergistically enhance the overall performance of various applications. This versatile approach opens promising possibilities in fields such as biomedicine, nanotechnology, and sensor development, providing a powerful platform for advanced research and technological innovations. In this review, the recent advancements in polymer-AuNPs synthesis and their applications in bioimaging will be covered. Prospects and challenges associated with polymer-AuNPs-based bioimaging agents in preclinical and clinical investigations will be discussed.

## 1. Introduction

**Figure 1:**
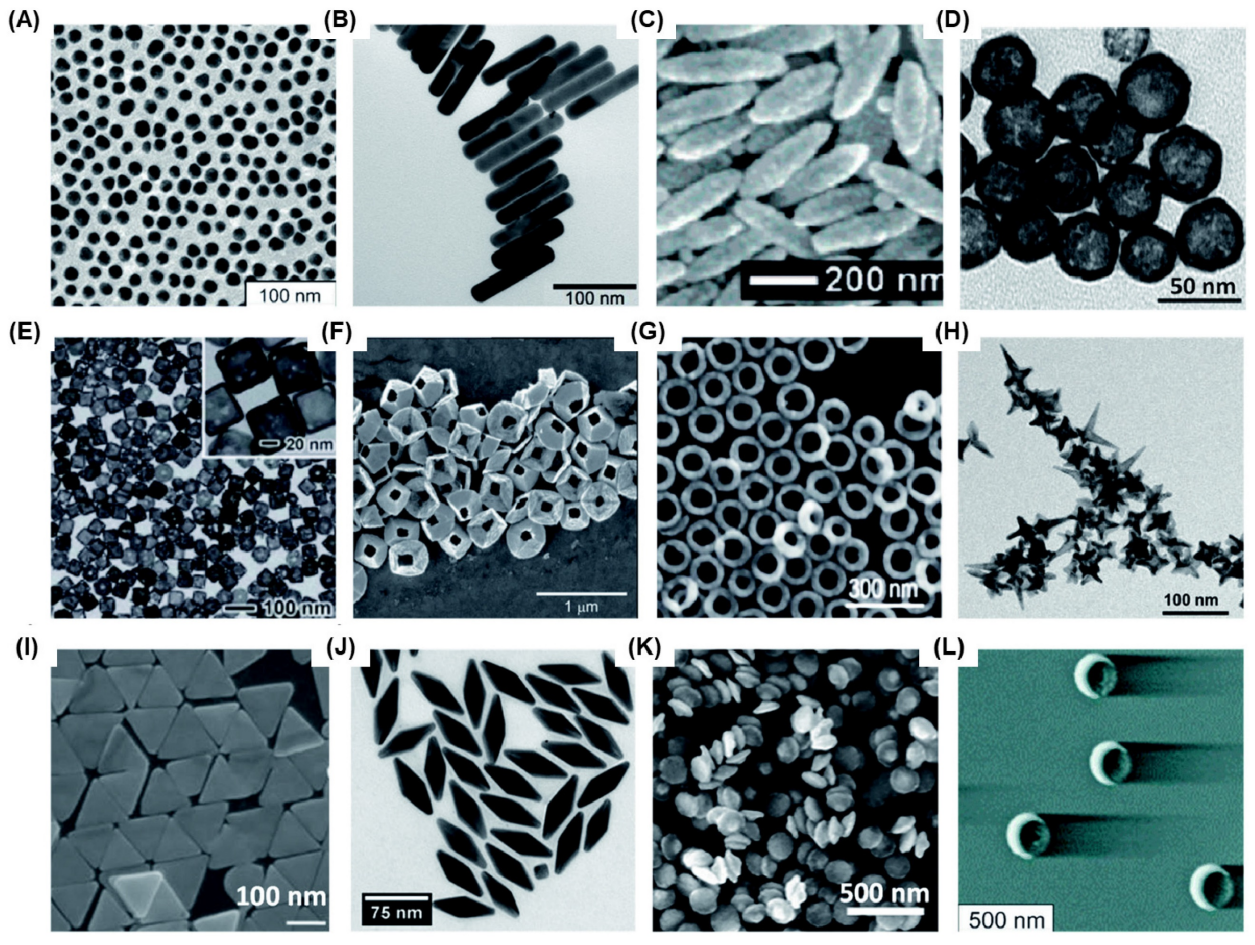
Various Au nanostructures synthesised for bioimaging applications. (A) AuNS (16 nm) (B) AuNR (C) Au nanorice (D) AuNSh (E) AuNC (F) tipless AuNPy (G) AuNRg (H) AuNSt (I) AuNPr (J) AuNBP (K) AuND and (L) AuNCr. Adapted with permission from 5, 7, 9, 13, 14, 17, 18, 21, 24, 25, 27, 29.

Bioimaging has emerged as an increasingly crucial technique for disease diagnostics and management, owing to its non-invasiveness, near real-time feedback, high accuracy, and reliability 1, 2. Optical imaging, which provides high-resolution images without the need for radioactive contrast agents, has been further strengthened by advancements in nanotechnology 3, 4. This has significantly enhanced its sensitivity, contrast, specificity, and multiplexing capabilities. Among the diverse range of nanotechnological tools investigated for optical bioimaging, gold nanoparticles (AuNPs) stand out due to their ease of fabrication, chemical stability, remarkable biocompatibility, and versatile optical properties. By manipulating the shape and size of AuNPs, various optical properties such as localized surface plasmon resonance (LSPR), the ratio between light absorption and scattering coefficients, surface-enhanced Raman scattering (SERS), fluorescence, and more can be finely tuned. Over time, researchers have successfully fabricated numerous Au nanostructures, including nanospheres (AuNS)^5^, nanorods (AuNR) 6-8, nanoshells (AuNSh) 9, 10, nanoprisms (AuNPr) 11-13, nanopyramids (AuNPy) 14, nanobipyramids (AuNBP)^15^-^17^, nanocages (AuNC) 18, 19, nanorings (AuNRg) 19, 20, nanodisks (AuND) 21, 22, nanostars (AuNSt) 23, 24, nanorice 25, nanobowls 26, nanocrescents (AuNCr) 27-29, and others which possess unique optical properties (Figure 1).

The utilization of diverse surface chemistry is crucial in various applications. When it comes to modifying AuNPs, one effective strategy is employing polymers, which has shown significant enhancements in biomedical applications 30, 31. Polymer modification of AuNPs not only improves their stability and dispersity but also expands their functionality. In nanomedicine, the presence of polymer shells around the Au core preserves its optical properties, allowing responses to internal or external stimuli like enzymes, pH, temperature, or light. For therapeutic purposes, the modulation of processes like high loading and controlled release of active pharmaceuticals from nanocarriers is crucial, and polymers prove to be instrumental in achieving these goals. Additionally, polymers can be employed in diverse bioimaging studies 32, 33. Although numerous studies have investigated the biomedical applications of gold-polymer nanoparticles, this review will specifically concentrate on exploring the potential of gold-polymer nanocomposites in the field of bioimaging, which presents a fertile ground for further investigation.

## 2. Gold or gold-polymer nanoparticles synthesis

The bottom-up chemical approach is commonly used for synthesizing AuNPs, where precise control over their shape, size, and stability is ensured. Faraday's method, which involves reducing NaAuCl_4_ with a phosphorus/carbon disulfide solution, is considered the first technique for synthesizing colloidal AuNPs. This method yields ruby-colored colloidal AuNPs with an average particle size of 6 ± 2 nm 34. Another method, known as the Turkevich method, utilizes sodium citrate as a reducing and stabilizing agent under reflux conditions to produce AuNPs with good dispersity 35. Chemical reduction using NaBH_4_ and seed-mediated multistep methods are also employed to synthesize AuNPs with different sizes and shapes 36-38. The seed-mediated method involves the formation of Au(III)-CTA complex, reduction of Au(III) using NaBH_4_ or citrate to produce Au seeds, and subsequent growth using AgNO_3_, and ascorbic acid to create nanostructures like AuNPs, AuNRs, AuNSs, AuSTs, AuCGs, etc. 39. In recent years, various other synthesis methods such as interfacial synthesis 40, laser ablation 41, arc discharge 42, microwave 43, electrochemical 44, and biological [45]and green chemistry 46 approaches have been investigated for AuNP synthesis.

**Figure 2:**
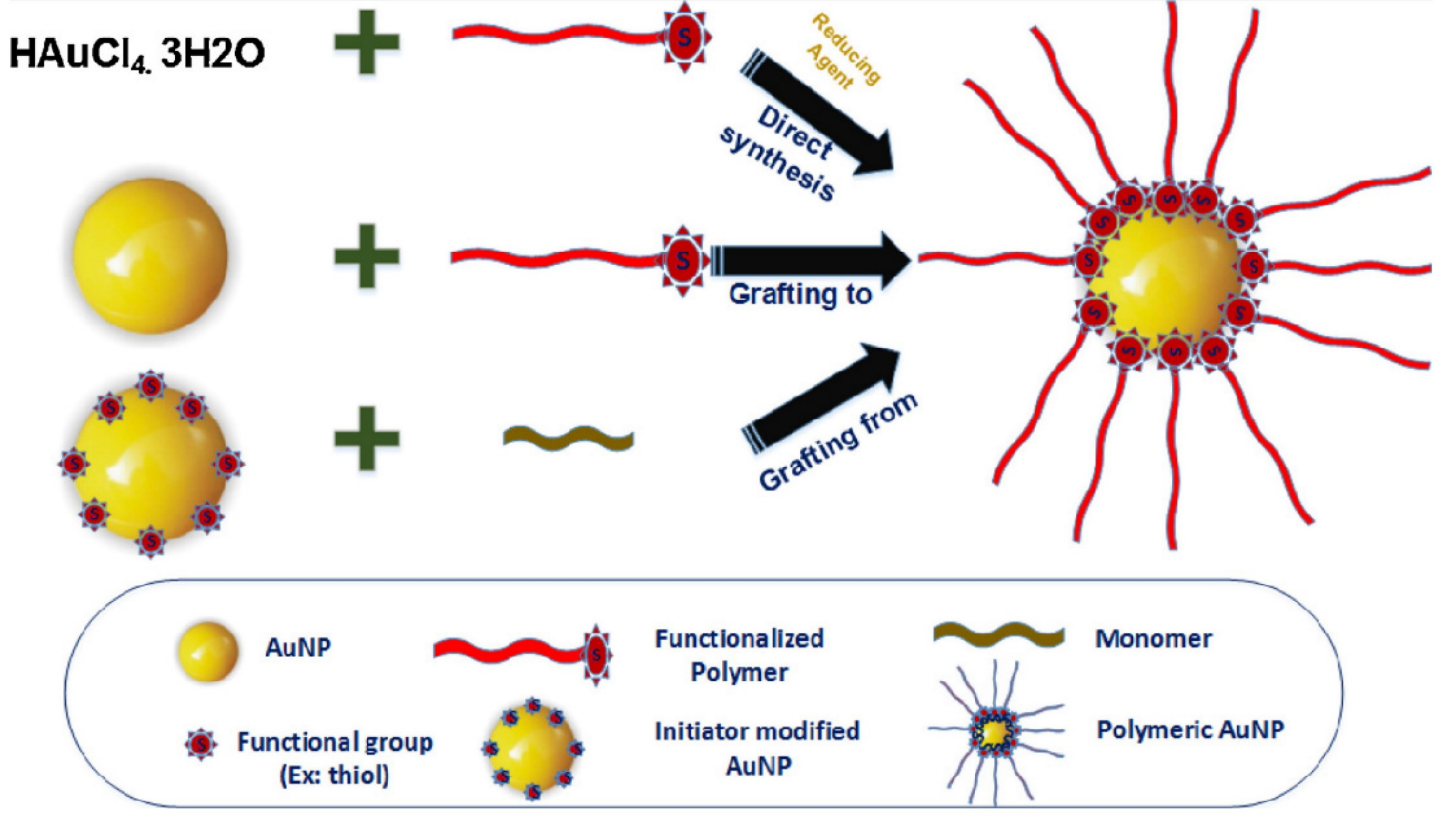
The diverse synthetic approaches employed in the production of polymer-encapsulated gold nanoparticles. Adapted with permission from 50.

The stability and toxicity of bare AuNPs pose significant challenges for their direct use in biomedical applications. To overcome this, surfactants or functionalized polymers are employed to achieve electrostatic or steric stabilization and prevent aggregation of bare AuNPs. Polymers with an opposite charge to that of AuNPs are physisorbed on the surface to create a passive layer, minimizing aggregation tendency 47. The direct synthesis approach involves reducing HAuCl_4_ in the presence of sulfur or amino-terminated polymers to obtain hybrid polymer AuNPs in a one-step process 48, 49. Covalent bond formation between AuNPs and various polymers is currently employed, with techniques falling into categories such as (i) graft-from, (ii) graft-to, and (iii) grafting-through as shown in Figure 2 50.

In the graft-from approach, polymerization initiators are first immobilized on AuNPs, and then polymer chains grow through the reverse addition-fragmentation chain transfer process. In the graft-to method, polymers with end functionalities, such as thiols or amines, are covalently linked to the surface functionalities of AuNPs. The grafting-through approach involves anchoring polymerizable groups on the NP surface, initiating polymerization in a solution containing monomers, initiators, and modified NPs acting as cross-linking agents 51. Post-modification methods involve conjugating polymers like biopolymers or DNA to the surface of pre-synthesized AuNPs, but this method suffers from low polymer loading efficiency and unintended adsorption through polymer functionalities 49, 52. Stabilizing and reducing agents like polystyrene, polyethyleneimine, poly(acryloylamino-phenyl arsenic acid), and xanthan gum are used for Au(III) to achieve desired sizes of polymer AuNPs, with variations achieved through different molar mass polyethyleneimine polymers and the number of branching units 53, 54.To ensure successful bioimaging applications, it is vital to maintain the stability of these nanocomposites while preserving the gold's SPR properties. The utilization of Au-polymer composites offers several advantages, such as their chemical inertness, retention of gold's SPR properties, reduced cytotoxicity, and stability in body fluids. These nanomaterials can serve as contrast agents in various bioimaging studies, which can be categorized based on the imaging tools employed.

Polymer stabilization of gold nanoparticles stands out as the preferred choice for biomedical applications, surpassing silica or amino-based stabilizations. These polymer coatings not only ensure exceptional biocompatibility but also enable precise surface engineering to facilitate targeted drug delivery, imaging, and cellular interactions 50. Their unique combination of flexibility, corrosion resistance, and anti-fouling properties makes them indispensable in maintaining nanoparticle integrity within intricate biological systems. AuNPs can be stabilized by silica shell or by amine coatings on their surface 55, 56. But silica and amine-coated gold nanoparticles have notable disadvantages compared to their polymer-coated counterparts for biomedical applications. Silica coatings can be relatively rigid, which can limit their adaptability within biological systems, potentially causing discomfort or tissue damage 57. Additionally, the thickness of silica coatings may hinder efficient drug loading or targeted cellular interactions 58, 59. Amino coatings may lack the versatility and tunability offered by polymers, making it challenging to fine-tune their surface properties for specific biomedical tasks. Additionally, amine modifications can sometimes lead to nonspecific interactions. Furthermore, both silica and amino coatings may have limitations in terms of biocompatibility, potentially triggering immune responses or adverse reactions 33. In contrast, polymer-coated gold nanoparticles offer a more flexible, customizable, and biocompatible solution, making them the preferred choice for diverse and sensitive biomedical applications 50.

## 3. Bioimaging applications of polymers and polymer nanoparticles

In recent times, the fusion of polymer chemistry with imaging science has ushered in a new era of polymer-based bioimaging probes tailored for disease diagnosis and treatment. The advancements in modern polymer chemistry have given rise to a diverse array of biocompatible polymer structures, such as dendrimers, multivalent polymers, branched polymers, graft polymers, and block polymers 60-62. These polymers, characterized by their precisely defined chemical compositions, have opened up avenues for the creation of innovative bioimaging probes that were previously unattainable through conventional small molecule-based methods. Polymeric bioimaging probes offer distinct advantages, including extended plasma half-lives, improved stability, reduced toxicity, enhanced targeting capabilities, and diminished nonspecific binding. These attributes enable the development of highly specific and amplified imaging probes capable of effectively distinguishing target areas from background in vivo.

Various polymers, such as polyethylene glycol (PEG), poly(D,L-lactic acid), poly(D,L-glycolic acid), and poly(ɛ-caprolactone), have already received clinical approval for use in large-scale formulations 63. Researchers have extensively explored polymer-based platforms for cancer therapy due to their numerous advantages 64. In particular, polymeric nanoparticles have demonstrated the ability to enhance treatment efficacy compared to free drugs 65. This improvement stems from their capacity to better encapsulate and deliver drugs, extend the time they circulate in the bloodstream, and release them in a sustained or controlled manner. Additionally, polymeric nanoparticles can target specific disease sites either passively, by exploiting the enhanced permeability and retention (EPR) effect, or actively, by incorporating targeting molecules designed for specific receptors or cell surface ligands at the desired location.

In the realm of therapeutic polymeric nanoparticles, there has been significant progress, with researchers now integrating clinically used imaging techniques into these therapeutic carriers. These include magnetic resonance imaging (MRI) contrast agents, radioactive substances for imaging through positron emission tomography (PET) or single photon emission computed tomography (SPECT), fluorescent agents for fluorescence-based imaging, as well as nano/microbubbles for ultrasound imaging.

### 3.1. MRI imaging applications

Polymeric nanoparticles have been shown to be effective carriers of both superparamagnetic iron oxide (SPIO) and gadolinium (Gd) for MRI applications. For instance, amphiphilic block copolymers, like maleimide-PEG-poly(lactic acid), can encapsulate both SPIO and the chemotherapy drug doxorubicin (DOX). These copolymers self-assemble into nanoparticles with maleimide groups on the surface for attaching targeting peptides. In tumor-bearing mice, these nanoparticles showed increased tumor-specific accumulation and inhibited tumor growth 66. In another example, biodegradable PLGA encapsulated docetaxel and SPIO, demonstrating sustained drug release with no initial burst effect. When used on PC3 prostate cancer cells, these nanoparticles exhibited high intracellular iron concentration and strong contrast in T2-weighted MRI 67. Similarly, HER2-targeted PLGA-PEG nanoparticles containing MnFe_2_O_4_ and DOX were used to target breast cancer, providing ultrasensitive MRI detection and effective tumor growth retardation in vitro and in vivo 68. Various polymers, including Pluronic ® F-127, have also shown promise for creating stable nanotheranostic formulations for encapsulating SPIO-drug combinations^69^.

The primary T1 MRI contrast agent, gadolinium(III), enhances positive image contrast by increasing water protons' longitudinal relaxation rate. In T1-weighted images, fluid appears dark, water-based tissues are mid-gray, and fat-based tissues appear bright. Typically, Gd(III)-diethylenetriaminepentaacetic acid (Gd-DTPA) or Gd-tetraazacyclododecanetetraacetic acid (Gd-DOTA) is used in polymeric nanocarriers for Gd-based MRI contrast. Ye et al. conjugated Gd-DOTA to poly(L-glutamic acid) (PLGA) side chains, demonstrating MRI signal enhancement in a breast tumor model. They found that higher molecular weight polymers (87 kDa) resulted in prolonged blood circulation and increased tumor accumulation 70. Liao et al. created hybrid nanoparticles with a hydrophobic PLGA core and a hydrophilic Gd-DTPA folate-coated PEGylated liposome shell for MRI and targeted drug delivery, offering high DOX loading efficiency and T1 relaxivities for high-resolution MRI 71. Hong et al. combined a DOX-loaded liposomal core with an acid-sensitive poly(acrylic acid) polymer shell functionalized with Herceptin and Gd(III), showing a 120-fold increase in cellular uptake of Gd compared to Gd-DOTA, leading to significant T1 MRI contrast enhancement 72. Other polymers used in cancer theranostics for simultaneous MR imaging and drug delivery include N-(2-hydroxypropyl)methacrylamide (HPMA)-based copolymers conjugated with Gd and therapeutic drugs 73, as well as multi-arm star block copolymers 74.

### 3.2. Radionuclide imaging applications

In the realm of medical diagnostics, radionuclide imaging serves as a valuable tool for assessing the progression of diseases by scrutinizing cellular metabolism and bodily physiology. Unlike MRI, which relies on observable tissue changes, radionuclide imaging boasts high sensitivity and is free from constraints related to tissue penetration. This technique involves the introduction of radioisotopes like ^11^C, ^18^F, ^64^Cu, ^76^Br, ^99m^Tc, ^111^In, and ^90^Y into the body via intravenous or oral administration. Subsequently, gamma cameras are employed to capture and generate images based on the radiation emitted by these internalized radionuclides. Numerous studies have extensively explored a range of radionuclide compounds, often in conjunction with various copolymers, with the ultimate objective of creating a robust nano-delivery system. For instance, Mitra and their team have coupled ^99m^Tc and ^90^Y with HPMA, followed by further conjugation with the αvβ3 targeting peptide RGD4C 75. These specialized polymer-radionuclide conjugates have demonstrated an enhanced affinity for adhering to endothelial cells expressing αvβ3 receptors, in addition to exhibiting antitumor efficacy in a SCID mouse xenograft model of human prostate carcinoma.

Similarly, Lammers et al. have harnessed HPMA to encapsulate ^131^I along with antitumor agents like doxorubicin or gemcitabine, thereby investigating the dual imaging and therapeutic capabilities of drug- and radionuclide-loaded polymeric nanocarriers 76. These polymeric drug carriers have showcased prolonged circulation times and selective accumulation at the tumor site. The synergistic action of these two components has led to heightened therapeutic effectiveness against tumors. Consequently, the integration of chemotherapy and radiotherapy within a single nanocarrier emerges as a promising strategy for combatting solid tumors.

### 3.3. Fluorescence imaging applications

Fluorescence imaging is a cost-effective technique that provides excellent spatial resolution within the UV-near infrared (NIR) wavelength range, comparable to the sensitivity of radioisotopes employed in PET and SPECT. Nevertheless, it has its shortcomings, including limited tissue penetration, potential high noise, background interference from tissue scattering of visible photons, tissue autofluorescence, light absorption by proteins, and interference from water molecules. However, the adoption of NIR light for in vivo imaging addresses some of these challenges, allowing for tissue penetration of several centimeters and mitigating autofluorescence and tissue scattering issues.

In a study conducted by Santra et al., NIR fluorescent probes were integrated into hyperbranched polyhydroxyl polymeric nanoparticles, along with the apoptosis-inducing protein cytochrome c 77. These nanoparticles were specifically targeted using a folic acid ligand, resulting in improved uptake and therapeutic effectiveness against various human carcinoma cells in vitro. Additionally, they emitted photons for imaging through the excitation of encapsulated indocyanine green (ICG). Similarly, Quadir and colleagues designed core-multishell nanoparticles consisting of hyperbranched polyethyleneimine (PEI) conjugated to monomethyl PEG 78. These nanoparticles were used to encapsulate and transport three distinct antitumor drugs (DOX, methotrexate, and sodium ibandronate) along with indocyanine-based dye and Nile red. When administered to mice with F9 teratocarcinoma, the core-multishell nanoparticles exhibited significant contrast within the tumor tissues compared to free dye, six hours post-administration.

Hu et al. successfully synthesized multifunctional micelles that possessed both fluorescent imaging and drug delivery capabilities. These micelles were created through the co-assembly of DOX-conjugated monomethoxyl PEG-block-poly(L-lactide-co-mercaptoethanol) copolymer, rhodamine B-conjugated mPEG-b-p(LA-co-ME), and folic acid-conjugated PEG-b-PLA copolymer 79. In in vivo fluorescence imaging experiments involving mice with hepatocarcinomas, it was observed that folic acid-conjugated micelles had prolonged accumulation in tumor tissues and displayed enhanced antitumor efficacy compared to free DOX or a non-targeted micelle formulation.

**Figure 3:**
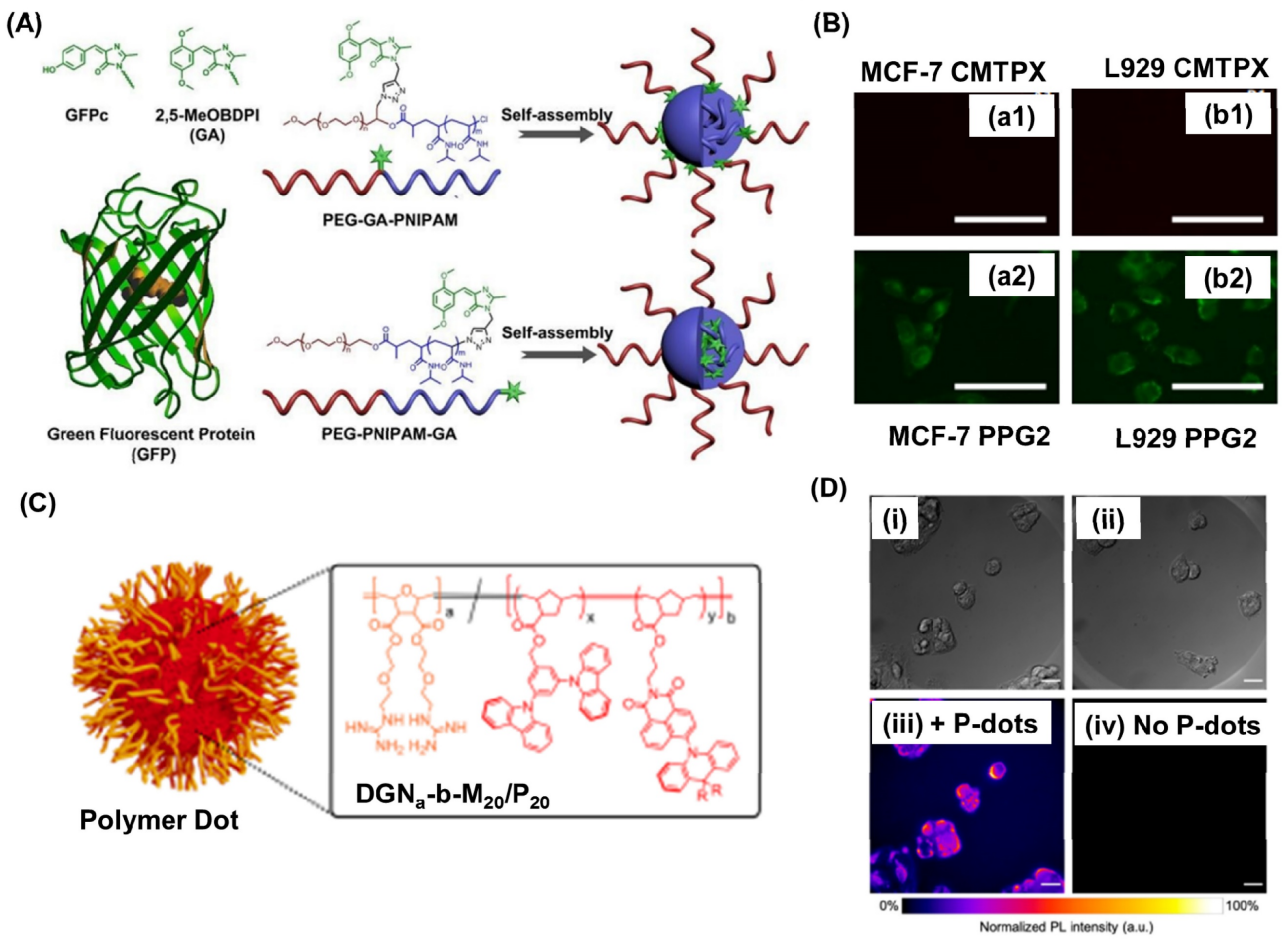
(A) Schematic representation for the fabrication of self-assembled polymers with GFP grafted at different positions. (B) Fluorescence imaging of MCF-7 cell lines (a1, a2) and L929 cells (b1, b2) using 71 μM of PEG-PNIPAM74-GA and 0.5 μM CellTrackerTM Red CMTPX for 30 min in DMEM with 10% FBS. Scale bar is 100 μm. (C) Cartoon representation for the water-soluble polymer dorts formed block polymers. Where R= CH3 or Ph. Orange color represents the block polymer part and red color represents the TADF block. (D) DIC images of HepG2 cells incubated with polymer dot and without polymer dots. Scale bar is 20 μm. Adapted with permission from 80, 81.

By employing click chemistry, Fan et al., ingeniously attached a self-restricted green fluorescence protein (GFP) chromophore analogue, inspired by the distinctive GFP structure, to both the middle and terminal positions of poly(ethylene glycol)-block-poly(N-isopropyl acrylamide) (PEG-b-PNIPAM), resulting in the synthesis of PEG-GA-PNIPAM and PEG-PNIPAM-GA (where GA represents MeOBDPI) (Figure 3A) 80. The results showed that increasing the length of the PNIPAM block enhanced fluorescence in water. PEG-PNIPAM74-GA exhibited higher fluorescence intensity than PEG-GA-PNIPAM106 in MCF-7 cells. Moreover, PEG-PNIPAM74-GA primarily localized in the cytoplasm and displayed easier cell entry in DMEM with 10% FBS compared to CellTracker™ Red CMTPX dye (Figure 3B). These findings suggest promising prospects for PEG-PNIPAM74-GA in living cell imaging. Christopherson et al. fabricated polymer dots exhibiting thermally activated delayed fluorescence (TADF) using ring opening metathesis reaction 81. Inspired by HIV's TAT protein, these polymer dots were fabricated using block copolymers with a hydrophilic guanidine-rich block and a rigid organic semiconductor block (Figure 3C). These polymeric nanoparticles quickly entered various cell types, maintained high cell viability at concentrations up to 25 mg mL^-1^, and had impressive quantum yields. They shielded the emitters from oxygen quenching, accumulating outside lysosomes to minimize degradation. In fixed cellular imaging, polymer dot-exposed cells showed superior signal-to-background ratios compared to controls, highlighting their potential for advanced imaging (Figure 3Di-iv).

### 3.4. Ultrasound and Photoacoustic Imaging applications

Polymeric microbubbles, with their stable characteristics in vivo and the ability to emit a robust non-linear signal when subjected to destructive ultrasound, offer exceptional promise among various microbubble types created from different membrane materials and core gases for enhanced ultrasound imaging sensitivity 82. Gao et al. employed block copolymers composed of PEG-poly(L-lactic acid) and PEG-polycaprolactone to create micelles that encapsulated doxorubicin 83. They introduced perfluoropentane (PFP) into the solution and subjected it to sonication, resulting in a mixture containing doxorubicin-loaded micelles and doxorubicin-loaded nanobubbles encapsulating PFP. In an in vivo breast cancer model, this mixture was observed to accumulate. Once accumulated, it transformed into microbubbles upon exposure to tumor-directed ultrasound. This transformation caused cavitation and subsequent collapse, leading to localized drug release and consequent tumor regression. Simultaneously, this approach enabled the molecular imaging of the nanobubbles. Haung et al. fabricated a triblock polymer system for stabilizing PFP for ultrasound imaging applications 84. The block polymer designed in such a way that it contains a fluorinated block for emulsification of PFCs, an alkene modified block for secondary thiol-ene cross-linking under UV radiation, and an oligoethylene glycol hydrophilic block.

Conducting polymer-based materials have found versatile applications in the field of photoacoustic imaging. One notable application is in the development of photoacoustic contrast agents, where polymers can be engineered to encapsulate light-absorbing molecules such as nanoparticles or organic dyes 85, 86. These polymer-based contrast agents enhance the photoacoustic signal, enabling more precise and sensitive imaging of biological tissues. Additionally, conducting polymers are utilized in the fabrication of photoacoustic sensors and transducers, contributing to the development of portable and high-resolution imaging devices. Their biocompatibility and tunable properties make polymers invaluable for various medical and preclinical applications of photoacoustic imaging, ranging from cancer detection and vascular imaging to monitoring physiological processes in real-time.

Pu and colleagues have introduced semiconducting polymer nanoparticles prepared from poly(cyclopentadithiophene-alt-benzothiadiazole) and poly(acenaphthothienopyrazine-alt-benzodithiophene) which absorb near-infrared light as innovative contrast agents for photoacoustic molecular imaging 87. These nanoparticles outperformed traditional agents like gold nanorods and single-walled carbon nanotubes, delivering enhanced signal strength and enabling real-time, in vivo imaging of reactive oxygen species. When combined with therapeutic agents, this platform holds significant potential as a robust theranostic tool. In another study ma et al. prepared a biodegradable semiconducting polymer from diketopyrrolopyrrole with excellent photoacousitc imaging application in vivo for the λ-carrageenan-induced arthritis mouse model 88. Polypyrrole based NPs were fabricated by Kesa et. al. and they studied the in vivo applications of these NPs for ultrasound and photoacoustic imaging applications on a mouse model 89. An intense photoacoustic signal was clearly observed in the anterior wall of the mouse heart after the NPs injection.

Thus from the existing literature reports polymers can enhance the bioimaging applications as a carrier for imaging agents or by modulating the surface and conjugation properties of polymers. Various polymer materials used for bioimaging applications are given in table 1.

## 4. Gold or gold-polymer nanoparticles for bioimaging

The first question that arises is how to clearly "visualize" the AuNP probes inside cells and various microscopy techniques provide viable choices 90, 91. Dark field (DF) microscopy is a valuable technique that takes advantage of the strong scattering signal from AuNPs when compared to cells and tissues, providing an effective means of detecting AuNPs within biological systems. Alternatively, differential interference contrast (DIC) microscopy offers another option, using two interference light beams to create contrast and enabling simultaneous imaging of nanoparticles along with cellular components like nuclei, vesicles, and microtubules. Interferometric scattering microscopy (iSCAT) is yet another approach, which relies on the interference between a reference light and light scattered by the specimen. This enables the generation of high interferometric contrast images with enhanced spatiotemporal resolution by eliminating static imaging background. Additionally, AuNPs' ability to absorb light can be harnessed for photothermal imaging, as it allows for the conversion of light absorbance into heat.

Molecular orientation and rotational motion are fundamental factors that play pivotal roles in various vital biological processes. Examples include the stepping motion of motor proteins, the self-rotation of F1-ATP synthase, Dynamin scission during clathrin-mediated endocytosis, and DNA twisting during polymerization and depolymerization. To study and understand these rotational dynamics in connection to essential biological events, researchers have turned to optically anisotropic AuNRs. These nanorods possess geometrically confined SPR peaks, which grants them unique properties as orientation and rotational probes. Their anisotropic absorption and scattering have been extensively harnessed with advanced optical microscopic techniques to investigate rotational dynamics linked to crucial biological occurrences. Consequently, advanced optical microscopic methods have gained widespread use in exploring the rotational behavior of AuNPs in these biological contexts.

In addition to visualizing the presence of AuNPs inside cells, another intriguing aspect is how these nanoparticles can enhance the detection of biological events. One interesting phenomenon related to AuNPs is their ability to enhance Raman signals for molecules 92. Raman spectroscopy is a powerful analytical technique that provides a unique chemical fingerprint of molecules within a biosystem 93. AuNPs can significantly amplify the Raman signal, leading to surface-enhanced Raman scattering (SERS) with over 10 orders of magnitude signal enhancement. This extraordinary sensitivity enables the detection of molecules at ultra-low concentrations (down to 10^-15^ M) and provides detailed information about chemical bonds and structures. Compared to other imaging methods, such as fluorescence, SERS offers sharper peaks, resulting in higher accuracy in detection. Consequently, SERS has gained attention for in vivo tumor diagnosis and therapy, expanding its potential for clinical imaging applications. Additionally, plasmon-enhanced fluorescence can be achieved on the surface of AuNPs due to the strong coupling between the fluorophore and the surface plasmon resonance (SPR) 94-96. This coupling effect can significantly alter the fluorescence emission, enhancing sensitivity and spatial resolution in fluorescence imaging. Moreover, the emerging field of fluorescence-emitting gold nanoclusters (AuNPs below 2-3 nm) enables direct fluorescence imaging of nanoparticles without the need for fluorophore conjugation 97.

In the field of optical microscopy, the imaging depth is often constrained to a few hundred micrometers within the tissue due to the significant scattering effect. To overcome this limitation and enhance contrast in in vivo optical imaging, AuNPs have gained considerable attention. Researchers have extensively investigated the use of AuNPs in various techniques, including two-photon or multi-photon luminescence, optical coherence tomography (OCT), and photoacoustic imaging (PAI). These methods offer the advantage of achieving tissue penetration depths ranging from millimeters to centimeters, enabling deeper imaging and analysis.

To prevent the nonspecific adsorption of plasma proteins and salts onto the surface of bare nanoparticles, which results in the formation of large aggregates, the direct application of AuNPs in living organisms leads to swift removal from the bloodstream as they are taken up by the reticular endothelial system, including macrophages in the liver and spleen. Hence, for the in vivo utilization of AuNPs, their surfaces need to be modified with antibiofouling agents like polyethylene glycol (PEG), and various biocompatible polypeptides or polymers 98. The extensive popularity of AuNP designs, characterized by their diverse range of sizes, shapes, surface functionalization, and assembly, has enabled their precise adaptation to meet the specific demands of various imaging applications. AuNPs decorated with dextrose and polysaccharide, such as chitosan, hyaluronic acid and fucoidan, play an important role for prolonged blood circulation, which is a key role for enhancing the contrasting ability of AuNPs 99, 100. Consequently, the utilization of AuNP-based probes for molecular imaging offers the flexibility to employ computed tomography (CT), fluorescence, optical imaging in various forms, photoacoustic imaging (PAI), magnetic resonance imaging (MRI), and other emerging techniques like surface-enhanced Raman scattering (SERS), dark field microscopy (DFM), optical coherence tomography (OCT).

### 4.1. Optical Imaging applications

AuNPs demonstrate a remarkable advantage in terms of scattered light intensity, ranging from approximately 10^5^ to 10^6^. This intensity surpasses that of commonly used fluorescent materials for imaging studies 101, 102. Furthermore, AuNPs exhibit exceptional stability and are not prone to photobleaching, making them an ideal choice as contrasting agents in bioimaging applications. Dark field confocal imaging (DFCI) and optical coherence tomography (OCT) are two widely employed techniques for backscattering AuNPs in bioimaging. DFCI is particularly effective in detecting even smaller AuNPs, including those below 10 nm in size. DFCI offers several advantages, such as generating bright spots from the scattering of AuNPs, surpassing the diffraction limits of lenses. It also provides high contrast and the ability to detect AuNPs with various sizes and shapes in a multiplex manner. To ensure stability and reduce cytotoxicity, biopolymers like proteins, peptides, and synthetic peptides can be employed to stabilize AuNPs.

**Figure 4:**
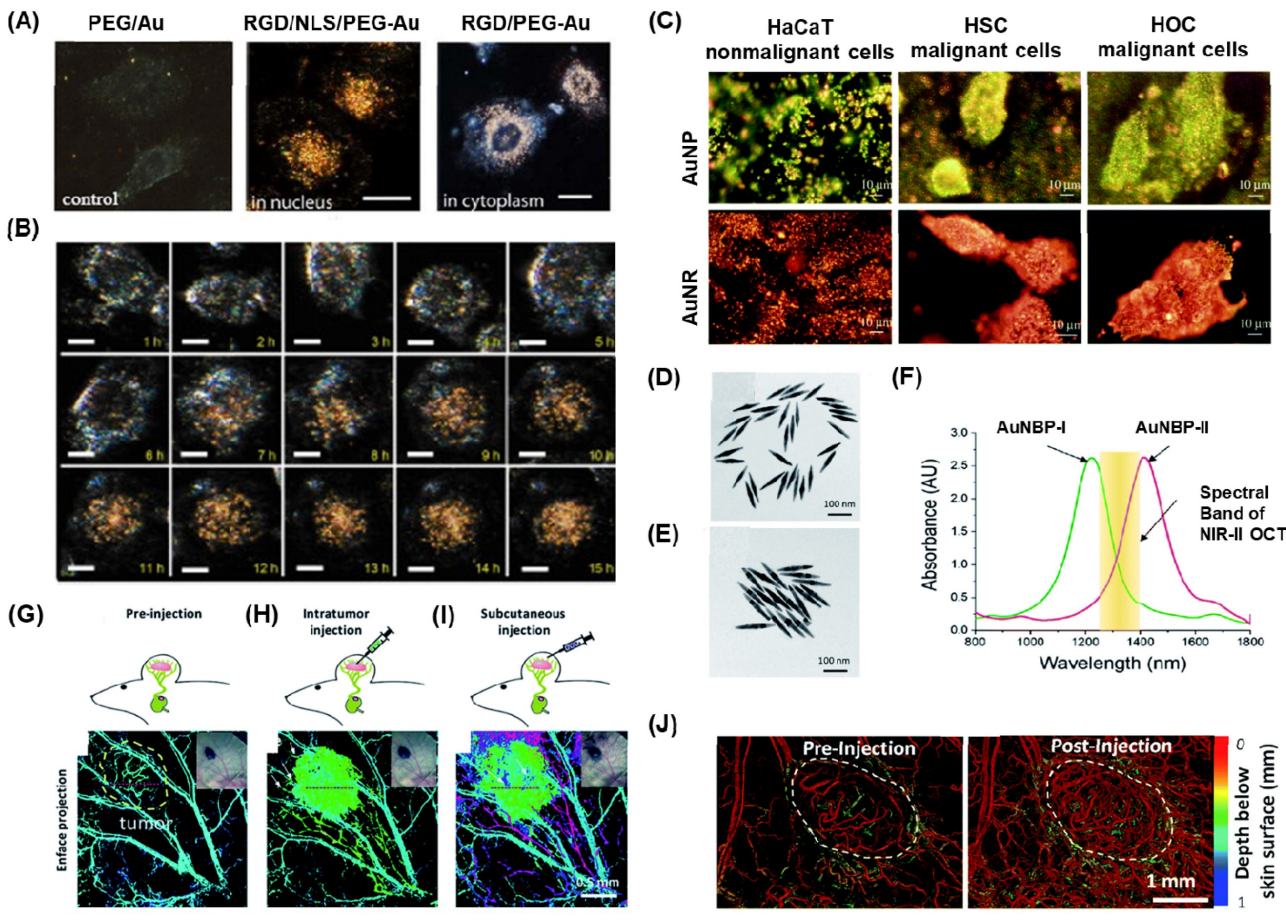
(A) Typical images of HSC-3 cancer cells after incubation with 0.1 nM PEG conjugated (left), RGD/NLS/PEG conjugated (middle) and RGD/PEG conjugated (right) Au NPs for overnight at 37°C (B) Time-dependent nuclear uptake of RGD/NLS/PEG-conjugated Au NPs the cells were immersed in cell culture medium at 37°C overnight in the presence of 0.05 nM RGD/NLS/PEG-conjugated Au NPs. Scale bar: 10μm. (C) Darkfield microscopic images of one nonmalignant and two cancer cells using spherical AuNPs and AuNR after incubating with cells for 30 min at room temperature. (D-E) TEM images for AuNBP with average sizes of 137 nm and 177 nm respectively. (F) Absorption spectroscopy details for AuNBP-I and II showing the applicability in the OCT windows in either side of the NIR region. (G) OCT showing the contrast in the endogenous vasculature in tumor (H) showing the additional contrast in the exogenous region after the injection of AuNBP-I (I) contrast developed by injection of AuNBP-II subcutaneously and allowing visualization of lymphatic system. (J) The OCT angiograms of a melanoma tumor implanted on a mouse ear before and after systematically injecting the NIR-II AuNPr. Adapted with permission from 11, 15, 103, 104.

Qian et al. demonstrated the preparation of thiolated poly(ethylene) glycol (PEG) (Mwt:5000), which was conjugated with nuclear location sequence (NLS) peptide CGGGPKKKRKVGG or cytoplasm region using RGD (RGDRGDRGDRGDPGC) via thiol chemistry 103. These peptide-conjugated AuNPs were employed as light contrasting agents, and the newly developed system allowed live cell imaging for up to 10 hours without any lag or bleaching (Figure 4A-B). This imaging system has been utilized to track the complete cell cycles of cancer cells from birth to division, monitor chromosome dynamics during cell mitosis, and observe the intracellular distribution of gold nanoparticles. Various types of AuNPs with different shapes, such as AuNS, AuNR, AUNC, and AuNSh, have also been utilized for cancer cell imaging using the dark field technique by functionalizing the surface of metal nanoparticles with various polymer bioconjugates 105-109.

In one of the studies, Huang et al. synthesized AuNP and AuNR conjugated with anti-epidermal growth factor receptor monoclonal antibodies and used this nanoparticle for differentiating nonmalignant epithelial cell line (HaCat) and two malignant oral epithelial cell lines (HOC 313 clone 8 and HSC 3) using DFCI (Figure 4C) 104. In order to make the overall surface charge of the nanoparticle system negatively charged for the physisorption of antibodies, the positively charged AuNR is coupled with poly(styrenesulfonate). Due to the specificity in the receptors on the surface of AuNP and AuNR, a strong scattered green to yellow and red light was observed in the DF images for AuNP and AuNR respectively. Laser ablation studies showed that the malignant cells need half the energy for the photothermal destruction as compared to the nonmalignant cells. Thus, this nanomaterial offered efficient cancer cell diagnosis and photothermal therapy applications.

Real-time tracking of virus infections is crucial and recently, Wan and colleagues developed an AuNPs-based scattering system for imaging respiratory syncytial virus and visualizing its infection of HEp-2 cells using the DFCI technique. To improve the specificity, the AuNPs were modified with streptavidin and the respiratory syncytial virus is labelled with biotin. The streptavidin-biotin interactions allowed specificity and despite its small size, the virus can be easily observed for an extended period without affecting its native activities by modifying it with streptavidin-AuNPs, which exhibit a strong light scattering 110.

The imaging technique known as OCT is based on interferometry and mainly utilizes NIR coherence light source to illuminate samples and detect the backscattered light. AuNPs have strong LSPR absorption, making them excellent candidates for enhancing back scattering at specific wavelengths. Various AuNPs with different anisotropic structures and dimensions, such as AuNR 7, 8, AuNSh 111, 112, AuNSt 113, 114, AuNC 21, 22, 115, have been employed for OCT.

PEG coated AuNBP and AuNPr showed improved stability and blood circulation time when the AuNPs used as contrasting agents 11, 15. Si et al. presented findings on the development of multiplexed contrast agents capable of simultaneously tracing distinct lymphatic flows originating from a melanoma tumor 15. They synthesized two sets of AuNBP with average lengths of 137 nm and 177 nm, resulting in narrow localized surface plasmon resonances (LSPRs) with peaks at 1225 nm and 1415 nm, respectively (Figure 4D-E). These peaks were positioned on either side of the center wavelength of the optical coherence tomography (OCT) source at 1320 nm (Figure 4F). In the pre-injection control image (Figure 4G), the endogenous OCT contrast facilitated visualization of the vasculature in the tumor region. Following intratumoral injection of AuNBP-I, the diffusion of the nanostructure allowed imaging of the tumor and lymphatic drainage. Subsequently, subcutaneous injection of AuNBP-II enabled further characterization of the lymphatic vessels, allowing visualization of peritumoral and intratumoral lymphatic vessels. Multiplexed imaging was made possible by utilizing a custom spectral analysis algorithm to deconvolve the spectral signal, providing exogenous spectral contrast for visualizing the separated lymphatic flows (Figure 4H-I). This dynamic imaging capability is particularly valuable in cases of lymph node metastasis. For instance, by labeling the nanostructures with different targeting biomolecules, multiplexed OCT can be employed as a preclinical tool to assess heterogeneous tumor cells expressing different receptors or to evaluate the diverse endogenous receptors present in the lymphatic system. In another study they demonstrated Au nanoprism significantly enhanced dynamic scattering signals in microvessels and improved OCTA sensitivity in skin tissue and melanoma tumors in live mice (Figure 3J) 11.

Recently, Tang and coworkers synthesized AuNPr coated with polyaniline to evaluate pH levels in the anterior of a fish eye 116. These nanoparticles exhibited an absorbance maximum at 760 nm in the pH range of 1-6. However, in the pH range of 8-14, the spectrum shifted, showing maxima at 600 nm and 900 nm, while the absorbance at 760 nm decreased significantly. This reversible switch in the spectra occurred within the pH 6-8 range and could be detected using OCT. Consequently, this approach could be utilized with OCT imaging to monitor pH changes in a phantom containing a tumor mimic, as well as in the eye of a crucian carp in an ex vivo setting. Although pH-sensitive OCT contrast agents are still in the early stages of development, the next crucial steps for in vivo imaging in animal models would involve incorporating targeting agents to regulate biodistribution and pharmacokinetics.

AuNRs have garnered significant research attention as OCT contrast agents because of their easily adjustable size and aspect ratio as well as their straightforward preparation method. Their plasmonic resonance can be finely tuned within the range of 600 to 1300 nm by modifying their aspect ratio, and the ratio of scattering to absorption cross-section can be altered by adjusting their size. SoRelle et al showed that AuNR dimensions showed 30-fold back scattering than the AuNR with small sizes. The Plasmonic absorption peaks for these different AuNRs used as spectral contrast agents and detected multiplexably by OCT 7, 8. These contrasting agents used for the retinal imaging and detecting circulating tumor cells 117, 118. Zerda et al used PEG-coated AuNR for OCT imaging in live mice eyes 7. The PEG improved the circulation time of AuNR and showed high-quality noninvasive contrast-enhanced imaging of OCT in living subjects.

Gold polymer nanomaterials play a pivotal role in revolutionizing optical bioimaging applications, showcasing their remarkable importance in this field. With their unique combination of properties, these nanomaterials offer unparalleled advantages for visualizing and understanding biological processes at the nanoscale. The incorporation of gold nanoparticles within polymer matrices allows for the precise tuning of their size, shape, and surface properties, enabling enhanced optical properties, such as increased brightness, photostability, and biocompatibility. These characteristics are crucial for efficient labeling of biological targets, facilitating real-time monitoring of cellular dynamics and interactions. The importance of gold polymer nanomaterials in optical bioimaging applications lies in their ability to bridge the gap between nanotechnology and biomedical sciences, paving the way for groundbreaking discoveries and advancements in diagnostics, therapeutics, and personalized medicine.

### 4.2. MRI imaging applications

MRI imaging, a non-invasive technique widely employed in clinical fields, allows for the visualization of tissue structure and function. In comparison to CT and X-ray, MRI offers enhanced contrast for soft tissue imaging 119, 120. The concept of MRI is closely connected to nuclear magnetic resonance. In the past, MRI machines experienced lengthy scanning periods, with the initial MRI image taking approximately 5 hours to scan. However, advancements in this field have significantly decreased the scanning time. Nowadays, modern MRI machines are capable of scanning the entire body in just a few minutes. The effectiveness of MRI relies heavily on two factors: longitudinal relaxation time (T1) and transverse relaxation time (T2). These values vary depending on the specific biological material being examined. To acquire MRI images, contrast enhancement agents containing gadolinium have been widely utilized due to their high paramagnetic nature 121, 122. However, these agents have been associated with reported toxicity by several researchers 123, 124. While Superparamagnetic iron oxide (SPIOs) has been utilized for specific tissue imaging studies, it has been noted that these SPIOs can generate reactive oxygen species, leading to tissue damage 125, 126.

In recent times, the significance of Au and heterogeneous metal oxide polymer nanocomposites has emerged as excellent alternatives for MRI contrasting agents 127, 128. Polymers are commonly used to enhance the stability of nanoparticles against aggregation and prolong their circulation time by disguising them from the immune system 129. The addition of polymers to the nanoparticle surface can have both positive and negative impacts on the relaxivity of a surface bound gadolinium (Gd) chelate. In most cases, the relaxivity decreases when a polymer surface coating is applied, as the polymer limits the access of water to the gadolinium centers 130. However, there are instances where the polymer restricts the motion of gadolinium chelates similarly to densely packed gadolinium chelates, resulting in an improvement in relaxivity 131. These conflicting effects on relaxivity have also been observed when attaching sugars to gold nanoparticles via linkers of varying lengths 132.

PEG serves as a crucial polymer in stabilizing gold nanoparticles and making them biocompatible. It has found extensive use in various applications, including the development of a novel 'smart' MRI contrast agent by Li et al. This agent demonstrates the ability to detect and sense tumor microenvironments based on their elevated acidity compared to healthy tissues 102. The functioning mechanism involves PEG units, which play a pivotal role. As the pH levels decrease in the tumor region, the PEG coating on the gold nanoparticle surface detaches, exposing alkyne and azide surface groups. Subsequently, a metal-free 'click' cycloaddition reaction occurs between these functional groups, leading to the formation of large nanoparticle aggregates. This aggregation results in an increase in relaxivity, thereby enhancing contrast in the tumor region. Consequently, this innovative nanoparticle design holds promise for guiding brain tumor surgery, where precise evaluation of tumor margins is of utmost importance to ensure successful outcomes.

**Figure 5:**
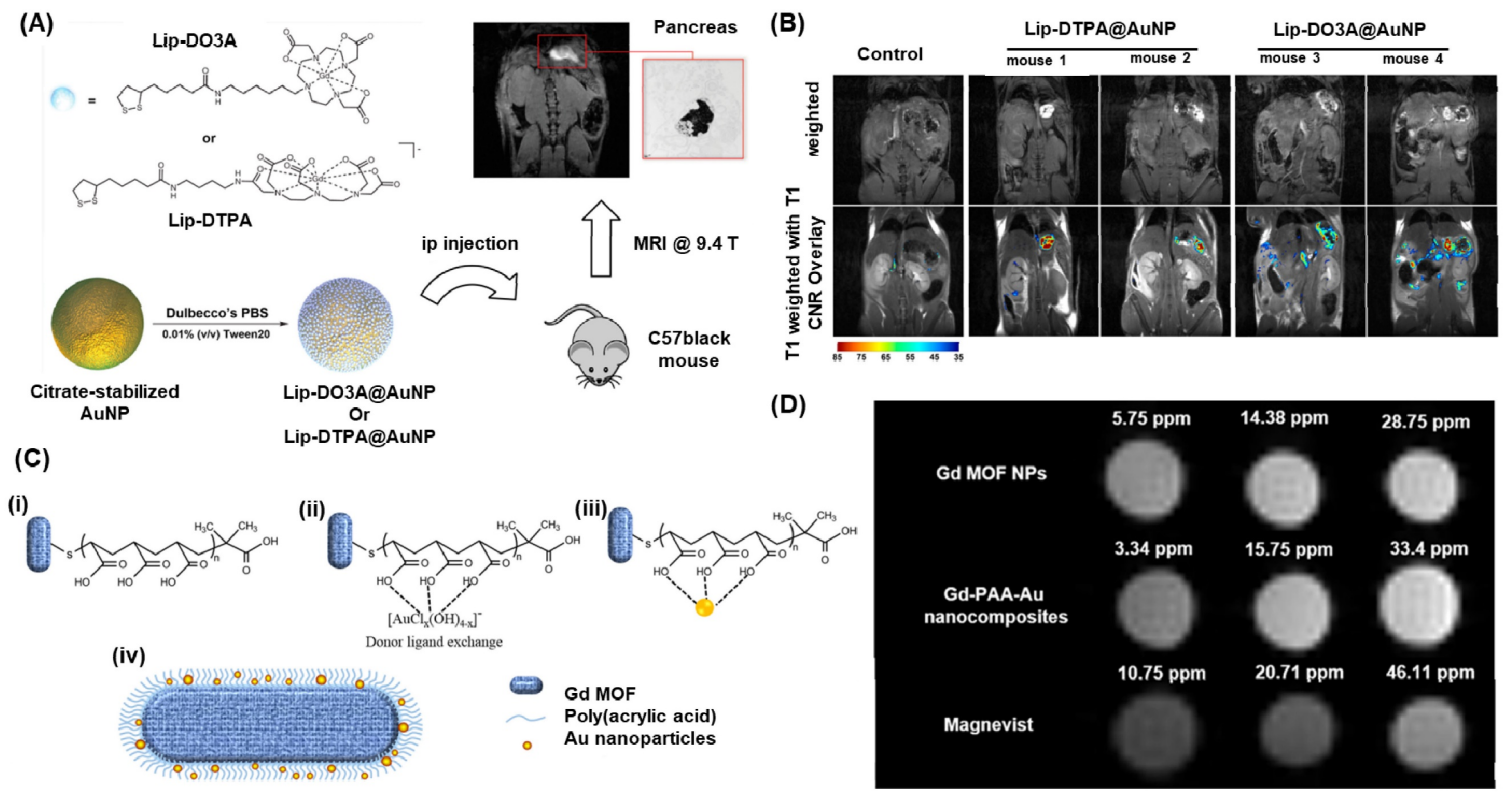
(A) Schematic representation for conjugation of Gd(III) Chelates (Lip-DO3A and Lip-DTPA) on to AuNPs and the MRI imaging application in C57 black wild-type mice. (B) T1-weighted flash images obtained at a magnetic field strength of 9.4 T from Lip-DTPA@AuNPs and Lip-DO3A@AuNPs. (C) Schematic for the fabricated Gadolinium metal-organic frame work with AuNPs. (i) deposition of PAA onto GdMOF nanostructures (ii) loading of Au(III) ions onto PAA-modified GdMOF nanostructures (iii) reduction of the Au(III) ions to produce AuNPs which get entrapped in PAA. (iv)Representation for the hybrid GdMOF-PAA-Au nanostructures for MRI applications. (D) T1-weighted MRI images of unmodified GdMOF nanoparticles, GdMOF-PAA-Au nanocomposite, and chelate-based Gd contrast agent (Magnevist) at various Gd concentrations in DIUF water. Adapted with permission from 134, 135.

The circulation time and biodistribution of AuNPs can be precisely modulated by utilizing small molecule PEG surface ligands, which can effectively adjust the surface charge of AuNPs. Remarkably, when administered systemically via intravenous (iv) or intraperitoneal (ip) routes, these AuNPs demonstrated a notable tendency to accumulate predominantly in the pancreas of athymic nude mice. This accumulation was attributed to the altered lymphatic clearance and intraperitoneal circulation following ip administration 133. Meade group presented a novel approach for the early detection of pancreatic adenocarcinoma by combining a dithiolane-Gd(III) complex with AuNPs (Figure 5A) 134. In their study, they successfully linked Gd-DO3A-C6 amine and Gd-DTPA-C4 amine to lipoic acid, which was then anchored onto the surface of AuNPs and showed a high Gd(III) payload. After injecting the solution into the peritoneal cavity and incubating it for 24 hours, we obtained MR images of the peritoneal cavity using standard T1-weighted FLASH scans at 9.4 T (n = 3 for Lip-DO3A@AuNP and Lip-DTPA@AuNP, respectively) (Figure 5B). We observed a significant enhancement in contrast for mice treated with Lip-Gd@AuNPs, enabling clear identification of the pancreas. Moreover, all subjects showed high contrast-to-noise ratios (CNRs). Consequently, these conjugates hold promise as potential candidates for the diagnosis and treatment of pancreatic diseases. In a similar vein, the Zhang group developed a probe called RGD@AuNPs-Gd99 mTc, consisting of c(RGDyC), Gd-, and 99 mTc-labeled AuNPs, varying in sizes (29, 51, and 80 nm) 136. They conducted evaluations of this probe's potential in vitro and in vivo for guided radiosensitization therapy, using MRI/SPECT as the guiding method. The findings illustrated that the RGD@AuNPs-Gd99 mTc with 29 nm AuNPs displayed the highest efficiency in vivo.

Feng et al. conducted a study in which they prepared a nanocomposite consisting of superparamagnetic Fe_3_O_4_/AuNR particles coated with polypyrrole. Combining the greater X-ray attenuation of Au, and photothermal property of polypyrrole along with contrasting property of Fe_3_O_4_ open up a nanocomposite material for various imaging applications 137. The synthesized nanocomposite exhibited remarkable magnetic properties and NIR optical absorbance, making it highly suitable for MRI and CT imaging. By irradiating the nanocomposite with an 808 nm laser, the solution containing it experienced a temperature increase of up to 35 ^O^C. In vitro photothermal treatment tests demonstrated the efficient eradication of cancer cells through the photothermal effects of the Au/PPY@Fe_3_O_4_ nanocomposites. This versatile nanocomposite holds promise for both multimode imaging and cancer treatment studies. Li et al. developed a polyethyleneimine-coated Fe_3_O_4_/Au nanostar shell for MR imaging application of mouse liver. The nanocomposite showed relatively high r2 relaxivity (146.07 mM^-1^ s^-1^) and good X-ray attenuation property 138.

Tain et al recently reported a dual-modal imaging contrast agent that combines Gadolinium metal-organic framework (GdMOF) with gold nanoparticles (AuNPs) (Figure 5C-D) 135. To create this agent, a bridge of poly(acrylic acid) is utilized to entrap the AuNPs within the GdMOF framework (Figure 5C (i-iii)). The qualitative analysis of the MRI results shows that as the Gd concentration increases (3.34, 15.75, and 33.4 ppm), both the unmodified GdMOF nanoparticles and the GdMOF-PAA-Au nanocomposite exhibit enhanced brightness (Figure 5D). Moreover, these findings indicate that both the unmodified GdMOF nanoparticles and the GdMOF-PAA-Au nanocomposite provide brighter images compared to the clinically employed chelate-based Gd contrast agent, Magnevist, even at lower Gd concentrations. These hybrid nanocomposites demonstrate exceptional performance in both MRI, with high longitudinal relaxivity, and CT imaging, making them a promising candidate for multimodal imaging probes.

To enhance the accuracy of diagnosing medical conditions, Zhang et al. prepared a contrast agent utilizing ultrasmall AuNPs and β-cyclodextrin (AuNP@CD) which provide numerous binding sites through host-guest interactions 139. Subsequently, the surface of AuNP@CD was modified with AD-PEG2000-PLL(Gd-DTPA), and folic acid was conjugated using bioorthogonal chemistry, enabling tumor cell targeting. In comparison to Magnevist (Gd-DTPA, r1 = 4.25 mM^-1^ s^-1^) commonly used in clinics, the AuNP nanocomposite exhibited enhanced longitudinal relaxivity (r1 = 19.47 mM^-1^ s^-1^) and demonstrated excellent biocompatibility. Moreover, in vivo studies demonstrated that the AuNP@CD-AD-PEG2000-PLL (DTPA-Gd) nanocomposite effectively penetrated tumors and accumulated in the tumor region, thereby enabling high-resolution MR imaging. These findings suggest that this AuNP nanocomposite holds potential as a tumor-targeted MRI contrast agent for diagnostic purposes. While several innovative approaches utilizing Au-polymer nanocomposites have been explored for MRI imaging, further detailed studies are necessary to assess their viability in human studies.

Thus gold polymer nanomaterials have emerged as a revolutionary tool in the field of MRI imaging, offering unprecedented opportunities for enhanced diagnostics and therapy. Gold polymer nanomaterials possess high stability and biocompatibility, making them suitable for prolonged circulation within the body. Their large surface area allows for efficient loading of contrast agents, enabling improved signal intensity and precise localization of target tissues. By harnessing the power of gold polymer nanomaterials, MRI imaging has witnessed a significant leap forward, leading to enhanced accuracy in disease detection, tracking therapeutic responses, and facilitating the development of personalized medicine.

### 4.3. Computer Tomography (CT) X-ray imaging applications

Gold nanocomposites have demonstrated remarkable applications in X-ray imaging due to their higher absorption coefficient compared to existing X-ray absorbing agents 140-142. The widely used iodine-based iopromide (Ultravist), which suffers from drawbacks such as renal toxicity, extensive catabolism, and high vascular permeability, can be effectively addressed by employing polymer gold nanocomposites 142, 143. Gold (Au) offers several advantages over iodine (I) in terms of its atomic number and absorption coefficient. With a higher atomic number and absorption coefficient, gold provides approximately 2.7 times greater contrast per unit weight compared to iodine. This makes it a preferable choice for imaging purposes. Specifically, imaging gold at 80-100 keV helps minimize interference from bone absorption and takes advantage of lower soft tissue absorption, leading to a reduced radiation dose for patients. Additionally, the higher molecular weight of gold nanoparticles allows for longer blood retention, enabling effective imaging after intravenous injection and potentially eliminating the need for invasive catheterization during diagnostic triage 144. Molecular imaging may also be feasible with gold nanoparticles, as each nanoparticle bound to a targeting agent can deliver a significant number of gold atoms to a specific receptor, thereby enhancing the imaging signal. While gold may be more expensive than iodine, its detectable amounts are low, and the substantial benefits make gold-mediated clinical radiography a viable option.

**Figure 6:**
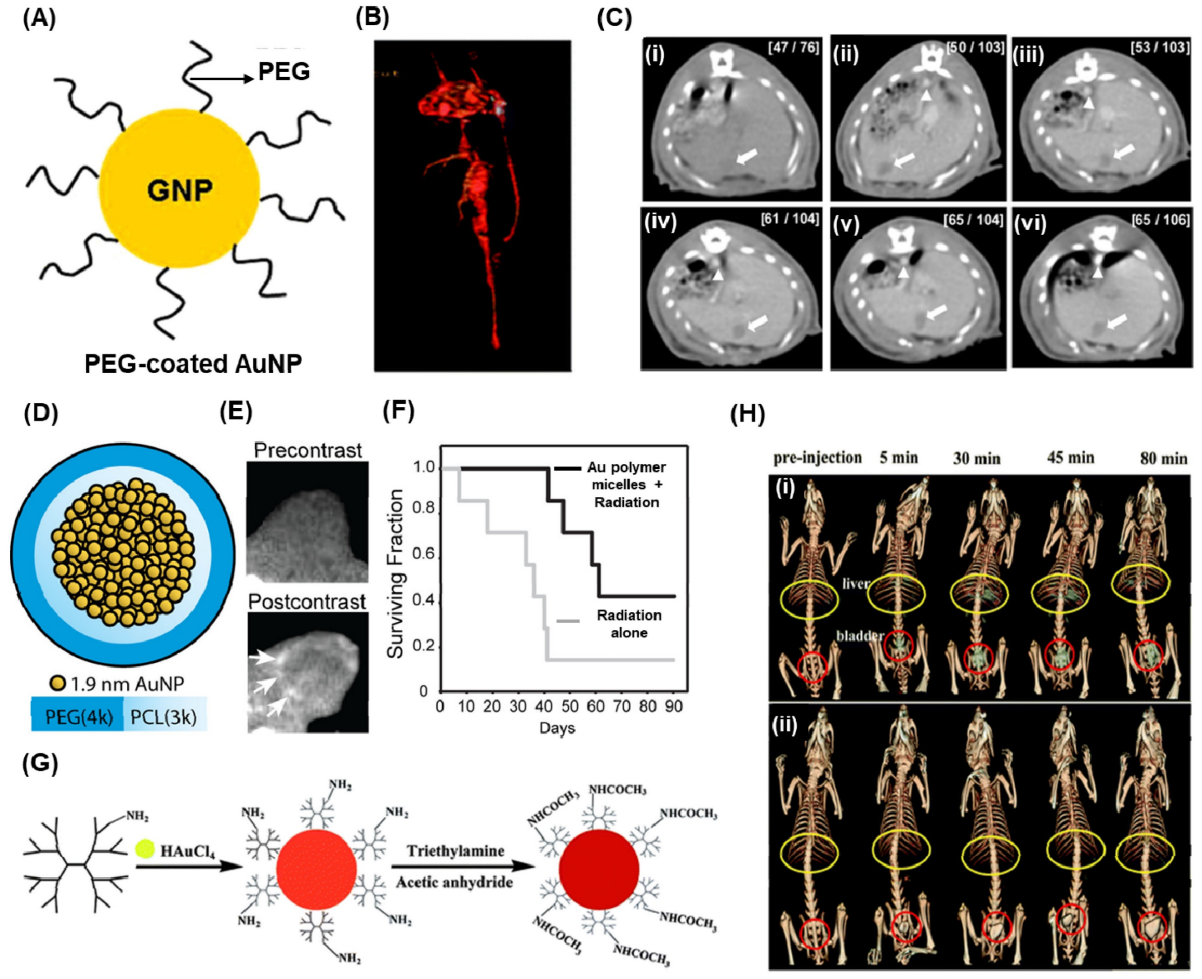
(A) Pictorial representation for PEG-coated AuNPs. (B) in vivo CT angiogram image of the heart and great vessels of Sprague-Dawley rat after 10 min of injection of PEG-coated AuNPs (140 mg/mL). (C) Serial CT images in a rat hepatoma model using 100 mg/mL of PEG-AuNPs at different times (i) 0 h (ii) 5 min, (iii) 1 h, (iv) 2 h, (v) 4 h, and (vi) 12 h after injection. (D) schematic of gold-loaded polymeric micelles (E) in vivo CT images of nude mice with HT1080 flank tumors prior and after 24 h of post injection. (F) Kaplan-Meier survival analysis for tumor bearing mice using gold-loaded polymeric micelles and radiation therapy. (G) schematic illustration for the synthesis of PAMAM reduced AuNPs. (H) in vivo CT images after intravenous injection of PAMAM-AuNPs (0.47 mmol Au per kg body weight (i) and (ii) CT image obtained for the same amount of Omnipaque in terms of iodine concentration. Adapted with permission from 142, 146, 147.

Hainfeld et al conducted a study demonstrating that the utilization of 1.9 nm-sized AuNPs enables effective CT-Xray imaging of tumors in mice 144. These injected nanoparticles were not detected in the bloodstream even after 24 hours, but they exhibited notable accumulation in the kidney, liver, tumor, and muscle just 15 minutes after injection. Due to their small size, these nanoparticles were efficiently cleared through renal excretion. In another study they proved that use of PEG and coupling the surface with anti-Her2 antibodies improved the microlocalisation of AuNPs (15 nm) in human breast cancer cells using CT imaging 145. In a recent development, Kim et al designed PEG-coated AuNPs (30 nm) with anti-biofouling properties, resulting in an extended systemic circulation half-life (Figure 6A-C) 142. The PEG-coated AuNPs exhibited a circulation time of over 4 hours, surpassing the performance of the commonly used iodine agent, iopromide, which circulates for less than 10 minutes. The feasibility of PEG-AuNPs as a contrasting agent for CT imaging was studied using blood pool imaging of rats after the intravenous injection of nanoparticles. As shown in figure 4B, the heart and great vessels can be distinguished on the PEG-AuNP-enhanced CT image with good contrast. A series of CT images for a rat liver with a hepatoma is studied at different times using PEG-AuNPs (Figure 6C (i-vi)). The hepatoma region and aorta are indicated by arrows and arrowheads, respectively. Initially, identifying the hepatoma in the pre-enhanced CT image (Figure 6C (i)) proved challenging. However, after the intravenous injection of AuNPs, a substantial (∼2-fold) enhancement in contrast between the hepatoma and the surrounding normal liver within 5 minutes could observed. Remarkably, this relative contrast difference remained consistent for up to 24 hours. These findings indicate that the developed PEG-AuNPs can serve as an effective CT contrast agent for hepatoma detection. Additionally, the AuNPs exhibited a prolonged circulation time as evidenced by the clear enhancement of the CT signal for the aorta (arrowhead) for at least 4 hours (Figure 6C (iv)).

The CT-X-ray imaging demonstrated a significant enhancement in contrast when using PEG-attached AuNPs along with antibodies. Reuveni et al. synthesized PEG-AuNPs and subsequently attached them with antibodies targeting the epidermal growth factor receptor (EGFR) 148. These engineered nanocomposites were investigated for their uptake in squamous cell carcinomas. The particles were administered via injection into the tail vein of mice with xenografted tumors, and the contrast levels were measured for both passively and actively targeted AuNPs within 0-3 hours. The results indicated similar contrast levels, but within 3-6 hours, the actively targeted AuNPs exhibited a twofold higher contrast. Another study by Kao et al. involved functionalizing PEGylated AuNPs with cetuximab, a drug that targets EGFR, to image human lung cancer cells (A-549) 149. CT imaging revealed that most of the particles accumulated in the liver and bladder, while the tumor remained visible up to 4 hours after injection.

Polymers serve a dual purpose in the context of AuNP utilization: reducing Au3^+^ and stabilizing AuNPs. Zhou et al. employed a PEGylated branched polyethyleneimine scaffold to synthesize AuNPs, which were then used in the bloodstream. When administered intravenously in a tumor model, these particles exhibited accumulation at the cancer site due to the robust EPR effect 150. CT imaging revealed high signal contrast when AuNPs incorporated into polymeric micelles. Zaki et al. focused on encapsulating 1.9 nm AuNPs within the hydrophobic core of micelles using the amphiphilic diblock copolymer poly(ethylene glycol)-b-poly(ε-capralactone) (Figure 6D)^146^. The resulting gold-loaded polymeric micelles demonstrated low polydispersity and varied hydrodynamic diameters ranging from 25 to 150 nm. These gold-loaded polymeric micelles were intravenously injected and provided long-lasting blood pool contrast for up to 24 hours, thereby enhancing the visualization of tumor margins using CT (Figure 6E). Additionally, the presence of gold nanoparticles in the micelles served as radiosensitizers, enhancing the response of tumors to radiation. Tumor-bearing mice treated with gold-loaded polymeric micelles enhanced radiation therapy exhibited a significant 1.7-fold improvement in median survival time compared to mice receiving radiation alone (Figure 6F). Li conducted a study where a gold nanocluster assembly was encapsulated by polyacrylic acid (PAA)/calcium phosphate. When administered intravenously in mice, these particles' fate was examined. Tumor tissue exhibited the highest CT signal and gold concentration, as determined by ICP atomic emission spectroscopy 151.

Uthaman et al. conducted a study focusing on the development of lymph node-targeted mannan-capped gold nanoparticles (M-AuNPs) as a contrast agent for CT imaging, utilizing green chemistry principles 152. Mannan act as both a reducing and stabilizing agent for AuNPs. The M-GNPs exhibited efficient uptake by antigen-presenting cells through the process of endocytosis mediated by mannose receptors. These nanoparticles, characterized by a spherical shape, had an average diameter of 9.18 ± 0.71 nm and displayed surface plasmon resonance absorption spectra, with the highest absorption occurring at 522 nm. Moreover, the M-AuNPs demonstrated a concentration-dependent X-ray attenuation property, reaching a maximum Hounsfield unit (HU) value of 303.2 ± 10.83. When administered locally, the M-GNPs significantly enhanced the X-ray contrast for imaging of popliteal lymph nodes. These findings provide evidence supporting the potential of M-AuNPs as targeted contrast agents for CT imaging, with specific biological applications.

A novel development involving Trastuzumab (TZ) multifunctional Fe_3_O_4_ coated Au NPs through PEG linking has emerged for targeted molecular computed tomography (CT) imaging 153. The TZ-PEG-Fe3O4@Au NPs were meticulously characterized and evaluated for their cytocompatibility, X-ray attenuation, and in vitro cell targeting ability. The results demonstrated that these synthesized nanoparticles, boasting a size below 100 nm, exhibited non-toxic properties within specific concentration ranges. Additionally, they displayed superior X-ray attenuation intensity when compared to iodine-based contrast agents at equivalent concentrations. Consequently, these targeted nanoparticles hold significant potential as contrast agents for molecular targeted CT imaging of cancer cells expressing the human epidermal growth factor receptor 2 (HER-2).

Dendrimers, a type of nano-sized polymer characterized by a well-defined composition and a branching tree-like structure, serve as a nanoplatform for the encapsulation and stabilization of various inorganic nanoparticles, such as metal or metal derivatives. Shi et al. introduced a convenient method for producing AuNPs stabilized by low-generation poly(amidoamine) (PAMAM) dendrimers, with the aim of utilizing them for in vivo computed tomography (CT) imaging applications 147. In this study, PAMAM dendrimers (G2) were used as stabilizers to form dendrimer stabilized AuNPs through a simple hydrothermal process, which were subsequently neutralized with acetic anhydride (Figure 5G). The resulting dendrimer stabilized AuNPs, with an Au core size of 5.6 nm, exhibited superior performance in CT imaging of major rat organs compared to Omnipaque, a clinical contrast agent (Figure 5H).

Qu et al. studied the receptor integrin, which is known to be overexpressed on certain tumor cells and tumor neovasculature, and its interaction with the Cyclo (Arg-Gly-Asp-D-Phe-Lys) (RGD) peptide 154. The approach involved conjugating the RGD peptide onto the surface of gold nanorods (AuNRs), resulting in RGD-AuNRs. These RGD-AuNRs have shown great potential in various applications, including tumor targeting and imaging using micro-CT imaging. To evaluate the effectiveness of designed nanomaterial experiments using integrin-positive U87 cells and integrin-negative HT-29 cells, both in vitro and in vivo were performed. The MTT assay and stability measurements demonstrated that the RGD conjugation eliminated cytotoxicity while improving biocompatibility and stability. Through dark-field imaging, the binding affinities and uptake abilities of RGD-AuNRs in U87 and HT-29 cells were monitored, and it revealed a higher specificity of RGD-AuNRs towards U87 cells. Additionally, the enhanced micro-CT imaging contrast achieved by intramuscular and subcutaneous injection highlighted the potential of RGD-AuNRs as contrast agents.

Thus, from the presented examples, gold polymer nanomaterials play a pivotal role in the field of CT imaging, offering immense significance and potential for enhancing diagnostic capabilities. These nanomaterials, consisting of gold nanoparticles with high atomic number of golds embedded within a polymer matrix, enables excellent X-ray attenuation and unique properties that make them highly desirable for medical imaging applications. By harnessing the power of gold polymer nanomaterials, CT imaging can achieve unprecedented levels of sensitivity, accuracy, and early disease detection, thus revolutionizing the field of medical diagnostics and paving the way for more effective and personalized patient care.

### 4.4. Photoacoustic Imaging applications

**Figure 7:**
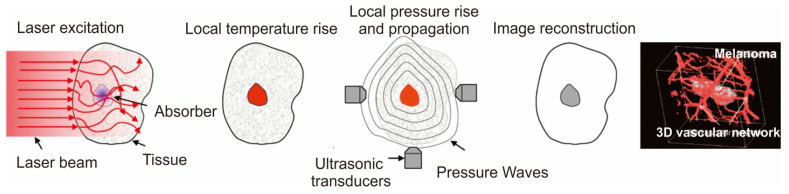
A schematic for the working principle and the high-resolution in vivo imaging performed in Melanoma using photoacoustic imaging. Adapted with permission from 157, 159.

Photoacoustic imaging (PAI), an increasingly popular biomedical imaging technique rooted in the photoacoustic effect enables cellular and tissue imaging utilizing both endogenous and exogenous contrasting agents 155, 156. Compared to fluorescence imaging, PAI exhibits superior spatial resolution and captures the molecular composition of diseased tissues with deep penetration. This is attributed to the minimal scattering of ultrasonic signals within tissues 157. Notably, the absence of ionizing radiation further elevates the prominence of PA imaging over alternative techniques. PA imaging applications can employ a range of contrasting agents, including both endogenous substances like melanin and hemoglobin, and exogenous substances like fluorophores, dyes, and nanomaterials. To enhance the imaging quality, exogenous contrasting agents are often preferred due to the low concentration of biomolecules 158. These agents work by absorbing light, causing thermoelastic expansion in tissues and generating acoustic signals. The resulting broadband sound waves are then detected by an ultrasound wave transducer, converted into PA signals, and ultimately transformed into images based on the time of signal arrival (Figure 7A) 85, 159. Among the various contrasting agents available, those based on gold nanoparticles (AuNPs) hold significant promise 158. The advantageous feature of AuNPs lies in their tunable surface plasmon resonance (SPR) property, which can be fine-tuned within the optical spectrum of 500-1100 nm, where minimal blood and tissue attenuation occurs. This characteristic enables the achievement of high contrast as the particles absorb within the biological window, a crucial criterion for successful PA imaging of deep tissues. Anisotropic AuNPs such as AuNRs 160, AuNSTs 161, AuNC 162, and AuNS 163 were commonly employed for PA imaging due to their high absorption rate. However, since spherical AuNPs typically exhibit absorption below 600 nm, polymer materials are commonly employed as capping agents to ensure stability and expand the absorption spectra window to higher wavelength regions for effective utilization of AuNPs in PA imaging.

Lu et al. conducted the synthesis of polyethylene glycol-coated hollow gold nanoparticles (PEG-HAuNS) with an average size of 40-50 nm in size. These PEG-HAuNS exhibited strong absorption resonance at 800 nm. These nanoparticles demonstrated significantly higher photoacoustic efficiency 9. When compared to photoacoustic tomography images based on the inherent optical contrast in nude mice, the utilization of PEG-coated hollow gold nanospheres as contrast agents resulted in clearer and more detailed visualization of brain vasculature. The images revealed brain blood vessels as small as approximately 100 micrometers in diameter. Initial findings indicated no acute toxicity in the liver, spleen, or kidneys of mice following a single imaging dose of PEG-HAuNS. Black et al investigated various PEG-modified gold nanostructures such as AuNS, AuNC, AuNR, and AuND for bioactivity analysis in an EMT6 breast cancer model 164. Studies revealed that both the AuNS and AuND were only observed on the surfaces of the tumors, whereas AuNR and AuNC were distributed throughout the tumors. Song et al formulated ultrasmall gold nanorod vesicles containing PEG, poly(lactic-co-glycolic acid) (PLGA), and AuNR, which exhibited excellent photoacoustic contrast ability and efficiency in photothermal cancer treatment 165. An outstanding characteristic of these nanocomposites is their prolonged circulation, significant tumor accumulation, and facile excretion from the body after targeted delivery and treatment. Sun et al. demonstrated the recent application of glycol-chitosan-coated gold nanoparticles (as a photoacoustic contrasting agent 166. Their findings revealed a significant enhancement in photoacoustic imaging of breast cancer cells. Notably, cell phantoms exhibited robust photoacoustic signals when incubated with Glycol-AuNPs for more than 3 hours, whereas PEG-AuNPs did not exhibit any increase in photoacoustic signal.

**Figure 8:**
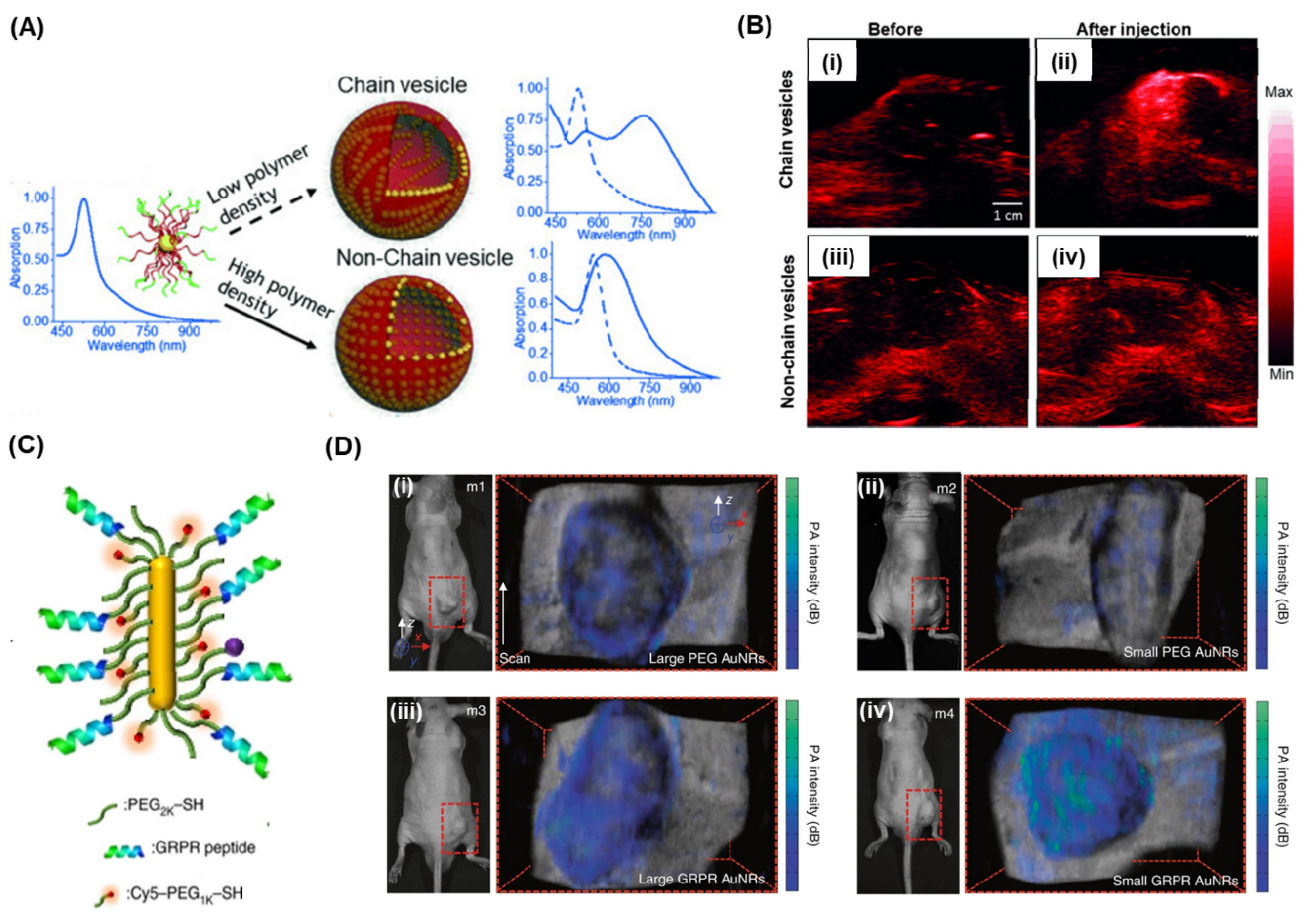
(A) Schematic and the absorption spectra for the formation of chain vesicles and non-chain vesicles from block polymer coated AuNPs. (B) In vivo 2D photoacoustic imaging of mouse tissue before and after the injection of chain vesicles (i, ii) or non-chain vesicles (iii, iv). (C) A schematic and (D) photographs of photoacoustic imaging for tumor-bearing mice from four different mice samples (m1-m4) using the larger and miniature AuNRs. (ii) and (iii) the PA imaging for the non-targeted larger and small AuNRs respectively whereas (iv) and (v) the PA imaging for the GRPR targeted larger and small AuNRs respectively. The colored maps represent the PA imaging signal intensity which are overlayed with the ultrasound images for the anatomical informationAdapted with permission from 168, 169.

Manivasagan et al. presented an innovative approach using doxorubicin-loaded fucoidan-capped gold nanoparticles (DOX-Fu AuNPs) as a multimodal system for drug delivery and PAI 167. Fucoidan served as both the capping and reducing agent for the gold nanoparticles, which were then conjugated with doxorubicin. The DOX-Fu-AuNPs were employed as a contrast agent for PAI to detect MDA-MB-231 cells noninvasively, exhibiting significantly enhanced photoacoustic signals due to optical scattering within the cells. Various techniques have been explored to improve the resolution of PAI. Yijing et al. introduced a method involving the folding of gold nanoparticle strings into plasmonic vesicles to enhance PAI (Figure 8A) 168. These hollow plasmonic vesicles, consisting of a string of gold nanoparticles, were synthesized using a stepwise self-assembly process. The resulting probes possessed tailored optical and physical properties, achieved by controlling the spatial arrangement of the gold nanoparticles. The experiments indicated that the vesicles exhibited strong absorption in the near-infrared (NIR) region, attributed to the presence of the gold nanoparticle string, resulting in highly efficient PAI (Figure 8B). AuNRs are commonly used as PAI agent due to their excellent surface plasmon resonance (SPR) and photothermal properties, which are influenced by their aspect ratios in terms of length and width, as well as their ease of synthesis. In a study by Chen et al., it was demonstrated that miniature-sized AuNRs (8±2nm by 49±8nm) exhibit light absorption in the near-infrared (NIR)-region and provide 3.5 times improved contrast for PA imaging compared to larger AuNRs (Figure 7C) 169. These miniature-sized AuNRs have an absorption peak at 1064 nm and are significantly smaller (approximately 5 to 11 times) than the commonly used AuNRs for photothermal and PA applications. In vivo experiments conducted on tumor tissues revealed a 4.5-fold enhancement in PA signal using these AuNRs, underscoring the importance of their structural characteristics in relation to optical absorption properties. Upon conjugation with GRPR-targeting peptides and Cy5 dyes, both the small and large AuNRs demonstrated target specificity. However, after 24 hours of injection, the non-targeted large AuNRs exhibited higher PA signal intensity than the small AuNRs, primarily due to tumor heterogeneity (Figure 8C; (ii-iii)). On the other hand, for the target-specific AuNRs, the smaller AuNRs displayed enhanced PA signal intensity compared to the larger AuNRs (Figure 8D; (iv-v)), highlighting the significance of their size in terms of target specificity and PA signal intensity.

Another promising strategy for cancer-selective detection using PAI is the targeting of cancer cell receptors through antibody conjugation. Mallidi et al. successfully conjugated anti-epidermal growth factor receptor (EGFR) antibodies to gold nanoparticles 170. Upon binding to the cell surface, the gold nanoparticles underwent molecule-specific aggregation, causing a red shift in their plasmon resonance frequency. The PAI results demonstrated high selectivity and sensitivity for tumor-mimicking gelatin implants in ex vivo mouse tissue.

### 4.5. Surface Enhanced Raman Scattering (SERS) Imaging applciations

SERS offers highly sensitive detection capabilities with significant enhancement (10^10^-10^14^ times) and superior reproducibility compared to alternative methods 171, 172. Recently, researchers have successfully utilized Raman reporter molecules attached to the surface of polymer nanocomposites containing AuNPs. These composites exhibit exceptional SERS enhancement and heightened sensitivity. Particularly, AuNP nanocomposites featuring rough metal surface tips and edges, known as "Hot-Spots," exhibit a remarkable amplification of Raman signal intensity compared to spherical AuNPs. To achieve well-dispersed metal nanoparticles, control interparticle distance, and manipulate the optical properties of these nanoparticles, polymers have emerged as a favorable choice for decorating meticulously patterned substrates for SERS 173-175. The incorporation of metal nanoparticles into polymer-based composites holds significant promise, offering multiple functionalities and the potential for cost-effective mass production.

Over the past two decades, the field of surface-enhanced Raman spectroscopy (SERS) has witnessed significant progress, due to the advancements in nanofabrication and Raman instrumentation, particularly confocal Raman microscopy. Combining digital imaging technology with Raman spectroscopy, confocal Raman microscopy enables the analysis of chemical composition, molecular structure, and spatial distribution of molecular components within a material, providing valuable insights into its microscale homogeneity. As early as 1975, Delhaye and Dhamelincour demonstrated the potential of combining Raman spectroscopy and mapping microscopy in their paper titled "Raman microprobe and microscope with laser excitation" 176. They presented the technique in detail, highlighting its applications in studying various materials such as rocks, plastics, composites, phases, inclusions, and defects in solids. They also emphasized its potential as a valuable tool for investigating chemical reactions in micro-samples and its extension to biological samples. Raman imaging involves collecting and analyzing thousands of spatially resolved spectra of compounds present in a specimen. By analyzing the intensities of diagnostic bands for each species, true maps depicting the spatial distribution of compounds can be generated without the need for stains, dyes, or contrast agents. This non-invasive characteristic makes Raman imaging highly advantageous for materials characterization, as minimal or no sample preparation is required for analyzing heterogeneous matrices. The versatility of this technique is evident in its applications across various fields, including pharmaceutical analysis, biology, biomedicine, label-free cell imaging, the food industry, threat detection, and fundamental research 93, 177.

Various polymer Au nanocomposites such as AuNSt 23, 24, AuNPy 14, AuNCr 28, 178, nanourchins 179, 180, AuNBP 181, 182, among others, have been employed to generate highly intense hot-spots for SERS applications. By carefully selecting the polymer matrix, polymer nanocomposites incorporating metal nanoparticles present several advantages in SERS applications. The choice of polymer allows for the development of a stimuli-responsive platform or a porous polymeric matrix that enhances the diffusion and entrapment of biomolecules during analysis. Within this framework, a significant goal in utilizing SERS substrates has been the detection and analysis of trace amounts of the target analyte, necessitating minimal specimen preparation.

In SERS bioimaging, these polymer-gold nanoparticles act as contrast agents, offering high sensitivity and specificity in detecting molecular targets within biological samples. By functionalizing the polymer shell with specific targeting ligands, such as antibodies or peptides, the nanoparticles can selectively bind to biomolecules or receptors of interest, enabling precise localization and visualization of specific cells or tissues. This targeted approach is particularly valuable in cancer research, where the nanoparticles can be engineered to seek out cancer cells and provide detailed information on their molecular composition and behavior. Additionally, the tunable plasmonic properties of gold nanoparticles allow for multiplexed imaging, where different types of nanoparticles with distinct Raman signatures can be used simultaneously to detect multiple biomolecules in a single experiment. This capability enhances the richness of the acquired data and enables a more comprehensive understanding of complex biological processes. Moreover, the biocompatible polymer shell ensures the nanoparticles' stability in biological environments, minimizing potential toxicity and ensuring prolonged circulation times in vivo. This stability, combined with the ability to load the polymer matrix with therapeutic agents or imaging probes, opens up exciting opportunities for theranostic applications, where the same nanoparticles can be used for both imaging and targeted drug delivery.

**Figure 9:**
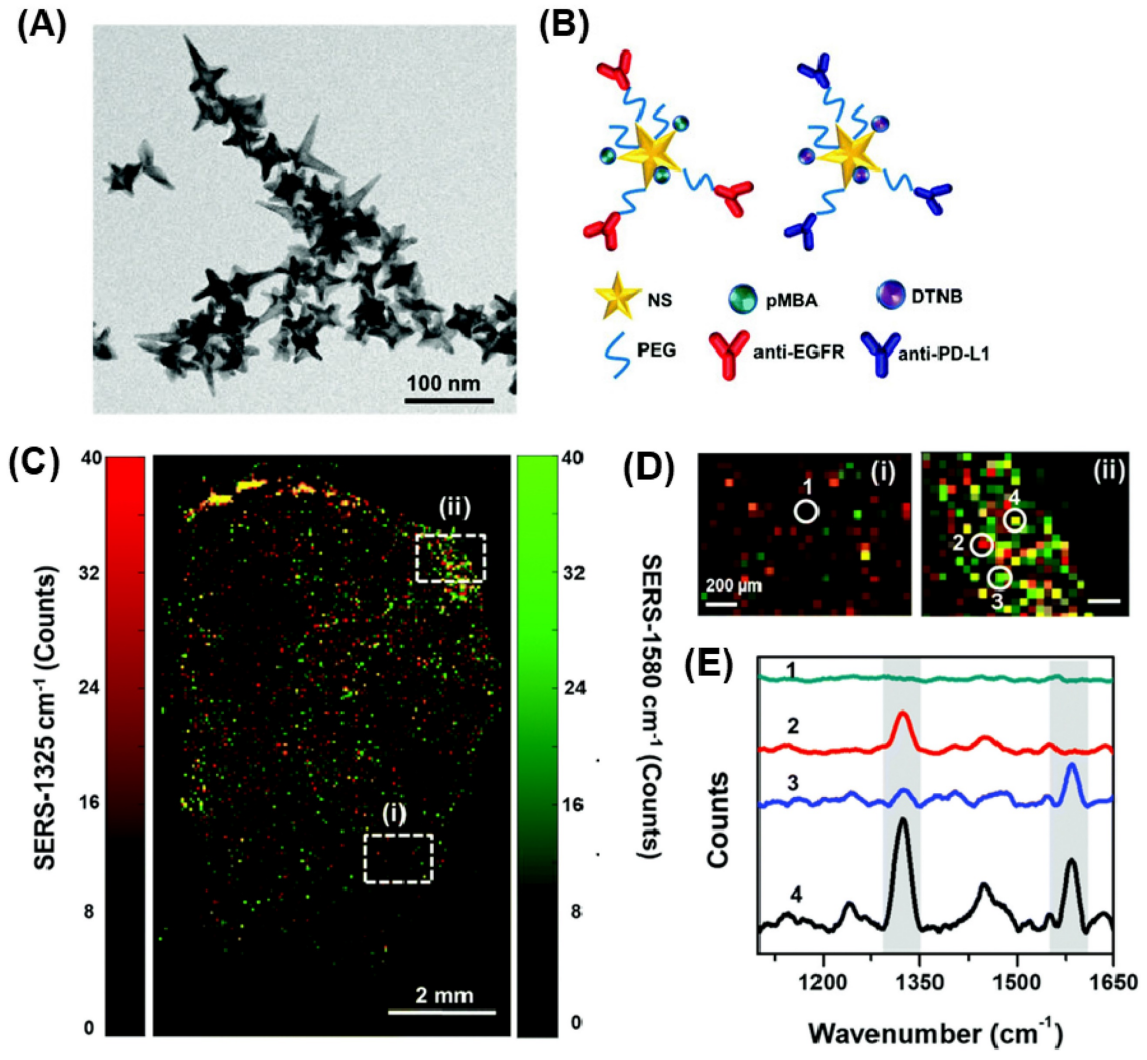
(A) Transmission electron microscopy (TEM) images revealing the morphology of AuNSt employed for surface-enhanced Raman scattering (SERS). (B) A schematic representation is depicted, showing AuNSt functionalized with two different Raman-active tag/targeting antibody pairs: pMAB paired with anti-EGFR and DTNB paired with anti-PD-L1. (C) Illustration demonstrates the SERS setup for multiplexed tumor immunoimaging. (D) SERS signals of DTNB (1325 cm⁻¹) and pMBA (1580 cm⁻¹) observed in ex vivo mouse breast cancer tissue. Subsection (i) displays a region of interest (ROI) with minimal AuNSt accumulation, while subsection (ii) exhibits an ROI with abundant AuNSt accumulation. (E) Corresponding Raman spectra of ROI (1)-(4) in (D), where (1) shows no AuNSt binding, (2) signifies high PD-L1 expression, (3) represents high EGFR expression, and (4) indicates elevated expressions of both PD-L1 and EGFR. Adapted with permission from 24.

Harmsen et al. demonstrated the exceptional sensitivity of a PEG-coated AuNSt-based SERS agent for precise detection of macroscopic malignant lesions and microscopic tumor invasions, by studying models of pancreatic cancer, prostate cancer, breast cancer, and sarcoma, including a human sarcoma xerograft model. Furthermore, this SERS-based technique offers the advantage of multiplex detection and imaging 183. Bardhan and colleagues recently reported the imaging of immune-biomarkers using AuNSt-based SERS imaging studies 23, 24. The synthesis of AuNSt carried out using biological buffer produced AuNSt with 100 nm size having ∼50-70 nm tip-to-tip dimension (Figure 9A). Raman tags and monoclonal antibodies specific to these biomarkers were conjugated onto AuNSt surface as shown in Figure 9B. Two sets of bioconjugated AuNSt were used in the experiment. The first set targeted PD-L1 and was labeled with the Raman tag 5,5-dithio-bis-(2-nitrobenzoic acid) (DTNB) and anti-PD-L1 monoclonal antibodies. The second set targeted EGFR and was labeled with the Raman tag para-mercaptobenzoic acid (pMBA) and anti-EGFR antibodies. Both DTNB and pMBA were attached to the surface of AuNS through a thiol group via covalent bonding. The monoclonal antibodies were conjugated to AuNSt using OPSS-PEG2000-NHS linkers, where the thiols on the orthopyridyl (OPSS) group bound to AuNSt, and the N-hydroxysuccinimide ester group formed an amide bond with the primary amines of the antibodies. Finally, a layer of thiolated-polyethylene glycol was added to the surface of AuNSt to ensure charge neutrality, minimize uptake by the mononuclear phagocytic system and enhance in vivo stability. In vivo SERS imaging was performed for the characteristic peaks of Raman tags in the AuNSt for the breast cancer tumor sections. The SERS spectra were subjected to several processing steps, including the removal of cosmic rays and subtraction of tissue autofluorescence. Subsequently, the intensities of DTNB and pMBA were represented as a color map, with each pixel assigned an RGB color. Specifically, DTNB was represented as red (1325 cm^-1^) and pMBA as green (1580 cm^-1^). The intensity map depicted in Figure 8C provides an overview of the localization of functionalized AuNSt, namely antiEGFR-pMBA-AuNS and antiPD-L1-DTNB-AuNS. By correlating the signal from each Raman tag to its respective targeted biomarkers, the map allows for qualitative assessment of biomarker status. To further investigate, specific regions of interest were identified within the spatially resolved Raman map of the tissue, enabling a closer examination of biomarker status (Figure 9C (i, ii)). High-magnification SERS maps (Figure 9D) offer cellular-level resolution of tumor areas exhibiting various biomarker profiles, such as PD-L1 richness (Figure 9Dii-2), EGFR richness (Figure 9Dii-3), co-enrichment of both biomarkers (Figure 8Dii-4), and potentially necrotic regions lacking AuNSt accumulation (Figure 9Di-1, no signal). The corresponding SERS spectra extracted from these regions of interest confirm the presence of PD-L1 and EGFR targeted AuNS distribution, as illustrated in Figure 9E.

Belhout et al. utilized polystyrene beads functionalized with lipoic acid, which were then loaded with 4-20% citrate capped AuNPs. The nanocomposite exhibited a notable redshift in the absorption spectra, attributed to the reduced average interparticle distance within the polymer matrix. Importantly, the magnitude of enhancement in SERS signal intensity was found to be influenced by the size of the AuNPs 184. In another study, Serrano-Montes et al. showed that hybrid nanomaterials from polystyrene beads and AuNSt showed improved SERS response and SERS bioimaging of living A549 cells using 4-mercapto pyridine as a Raman reporter 185.

Despite the high sensitivity of SERS probes, the Raman intensity experiences significant attenuation while passing through tissue due to scattering. Researchers sought to enhance tissue penetration by combining deep Raman spectroscopy with surface-enhanced, spatially offset Raman spectroscopy (SESORS), which resulted in a remarkable improvement, increasing the penetration depth from less than 5.5 mm to 25 mm thickness 186. To further reduce tissue scattering, the use of Raman reporters in the NIR range was explored, and gold nanostructures like AuNRs proved to be beneficial in this regard. Maltzahn et al. employed nanorods coated with SERS-active molecules, enabling unique in vivo distinction over a spectral sharp bandwidth of 6 nm in the NIR, much smaller than that of semiconductor quantum dots (QDs), organic fluorochromes, and Raleigh scattering nanoparticles. This platform found applications in SERS imaging and plasmonic photothermal therapy in mice 187. Additionally, Qian et al. utilized functionalized AuNRs conjugated with Raman markers for sentinel lymph node mapping and tumor targeting in mice. Moreover, these AuNRs facilitated the observation of their distribution and excretion in deep tissues through purely optical imaging in vivo 188.

In light of this, the selection of a polymer for a bioimaging application depends on the particular application, such as targeted imaging, drug delivery, in vivo or in vitro investigations, and the desired features such as biocompatibility, degradability, optical properties, etc., as well as the plasmonic properties of AuNPs. Table 2 provides an overview of the various Au-polymer based nanoparticles used for bioimaging applications and the mode of stabilization of polymers with AuNPs.

## 5. Clinical trials using AuNPs

Despite the enormous promise that AuNP-based nano theragnostics have showed in their early phases of research, they still have a long way to go before receiving clinical approval for use in patient therapy in a clinical setting. Precursor salts made of gold were once used to cure a variety of illnesses. For instance, in the early 1990s, rheumatoid arthritis was treated using ionic gold salts 189. However, once a more effective medicine with fewer side effects appeared, their use declined, highlighting the changing environment of medical therapies.

Currently, there are no clinically approved treatments based on AuNPs. While several nanoparticle-based drugs, devices, and diagnostic tools have received approval from regulatory agencies such as the USFDA and UKMHRA, none of them are AuNP-based as of the literature reports 190, 191. However, the NIH library's Clinicaltrials.gov database indicates that there are nine ongoing and completed clinical trials involving AuNP based therapeutics.

One notable study at Cardiff University is in Phase 1 of clinical trials (NCT02837094), where AuNPs are administered intradermally via microneedles to deliver immunotherapy with proinsulin-derived peptides for type 1 diabetes. Another study (NCT03020017) investigates the efficacy of NU-0129, a spherical nucleic acid AuNP targeting BCL2L12, a protein family member, to cross the blood-brain barrier in patients with recurrent glioblastoma multiforme or gliosarcoma, showing promising results for potential glioblastoma treatment 192. Additionally, a three-arm clinical trial (NCT01270139) is exploring the use of silica-coated AuNPs in treating stable angina, heart failure, atherosclerosis, and multivessel coronary artery diseases, with positive outcomes suggesting a promising future for gold nanoparticle-based therapies against atherosclerosis 193. Despite these promising findings, regulatory approval for clinical use will depend on demonstrating minimal side effects and optimized therapeutic efficacy for gold-based nanoparticles.

## 6. Conclusion and future prospective

In conclusion, polymer gold nanomaterials hold tremendous promise for bioimaging applications. The combination of the unique properties of gold nanoparticles and the versatility of polymers offers a range of benefits, making them excellent candidates for various imaging modalities. Over the years, extensive research has demonstrated the potential of these nanomaterials in improving imaging sensitivity, resolution, and biocompatibility, thereby advancing diagnostic and therapeutic strategies in medicine and biology.

Looking ahead, the field of polymer gold nanomaterials for bioimaging is likely to witness significant growth and innovation. Several key future prospects include:

i) Multimodal Imaging: Researchers will explore the integration of multiple imaging modalities into a single polymer gold nanomaterial, allowing simultaneous visualization of different aspects of biological processes. This will enhance the accuracy and depth of information obtained from imaging studies.

ii) Theranostics: The combination of imaging and therapeutic capabilities within a single nanoparticle, known as theranostics, will gain further attention. Polymer gold nanomaterials can serve as both imaging agents and drug delivery vehicles, enabling personalized medicine approaches.

iii) Targeted Imaging: Advancements in surface functionalization techniques will enable precise targeting of specific cells or tissues, enhancing the selectivity and efficiency of bioimaging. Targeted polymer gold nanomaterials could play a crucial role in early disease detection and monitoring treatment responses.

iv) In Vivo Applications: The translation of polymer gold nanomaterials from bench to bedside will be a major focus. Extensive preclinical and clinical studies will be necessary to evaluate their safety, biocompatibility, and long-term effects in living organisms.

### Challenges

Despite the promising potential, several challenges need to be addressed before polymer gold nanomaterials can be widely adopted in bioimaging applications:

i) Biocompatibility and Toxicity: Ensuring the biocompatibility of these nanomaterials and understanding their potential long-term toxicity in the human body is of utmost importance for clinical translation.

ii) Regulatory Hurdles: The regulatory pathway for nanomedicines, including polymer gold nanomaterials, is complex and requires meticulous evaluation to ensure safety and efficacy before widespread clinical use.

iii) Scalability and Manufacturing: Developing scalable and reproducible synthesis methods for polymer gold nanomaterials is essential for their practical application and commercialization.

iv) Stability and Storage: Maintaining the stability of these nanoparticles during storage and transportation is crucial to avoid aggregation and loss of imaging properties.

v) Cost-effectiveness: As with any new technology, cost considerations play a significant role in widespread adoption. Efforts to optimize production processes and reduce manufacturing costs will be vital.

vi) Image Interpretation: As the complexity of imaging data increases with multimodal and targeted approaches, appropriate image analysis tools and algorithms will be required for accurate interpretation and diagnosis.

Addressing these challenges will require collaboration between scientists, clinicians, regulatory bodies, and industry stakeholders to unlock the full potential of polymer gold nanomaterials for bioimaging applications, paving the way for improved disease diagnosis, treatment monitoring, and ultimately, better patient outcomes.

## Figures and Tables

**Table 1:** Bioimaging application of polymers

Polymer	Imaging modality	Type of cells	Ref
maleimide-PEG-PLGA+ SPIONS	MRI	In vivo	66
PLGA + SPIONS	MRI	PC3 prostate cancer	67
PLGA-PEG +MnFe_2_O_4_	MRI	Breast cancer	68
Pluronic ® F-127 +SPIONS	MRI		69
poly(L-glutamic acid + Gd-DOTA	MRI	breast tumor	70
poly(acrylic acid) + Gd(III)	MRI	-	72
PCL-*b*-P(OEGMA-*co*-AzPMA) + Gd-DOTA	MRI	Rat liver and kidney	74
HPMA + ^99m^Tc and ^90^Y	Radioisotope imaging	Human prostate carcinoma	75
HPMA + ^131^I	Radioisotope imaging	solid tumors	76
Polyhydroxyl polymeric NPs +ICG	Fluorescence imaging	human carcinoma cells	77
Polyethyleneimine (PEI) conjugated to monomethyl PEG +ICG	Fluorescence imaging	F9 teratocarcinoma	78
PEG-block-poly(L-lactide-co-mercaptoethanol) conjugated with Rhodamine B	Fluorescence imaging	Hepatocarcinomas	79
poly(ethylene glycol)-block-poly(N-isopropyl acrylamide +GFP	Fluorescence imaging	MCF-7 cells	80
Gaunidine block polymers	Fluorescence imaging	HepG2 cells	81
PEG-poly(L-lactic acid) +PFP	US	Breast cancer	83
poly(cyclopentadithiophene-alt-benzothiadiazole) NPs	PA	In vivo imaging of reactive oxygen species	87
Diketopyrrolopyrrole polymers	PA	λ-carrageenan-induced arthritis mouse model	88
Polypyrrole NPs	PA	Mouse heart	89

**Table 2:** List of gold-polymer nanoparticle systems studied for bioimaging modalities.

Nanoparticles	Polymer coating	Linkage	Imaging application	Technique	Ref
AuNBP	PEG	-SH-Au	Microvessels in skin tissue and melanoma tumors in live mice	OCTA	11
AuNPr	PEG	SH-Au	Lymph node in a live mouse	OCT	15
AuNPs	PEG	SH-Au	In vitro HSC-3 cancer cells	DFCI	103
AuNR	Poly(styrenesulfonate)	Electrostatic	HaCat, HOC 313 clone 8 and HSC 3	DFCI	104
AuNP/Fe_3_O_4_	PEG	-SH-Au	Pancreatic adenocarcinoma	MRI	134
AuNR/ Fe_3_O_4_	Polypyrrole	Electrostatic	Actin cytoskeleton	MRI/CT	137
AuNSt/ Fe_3_O_4_	Polyethyleneimine partially modified with PEG	-SH-Au	Rat liver and aorta	MRI/CT	138
AuNP	Polyethyleneimine	-NH_2_ -Au	Blood pool, lymph node and tumor in mice	CT	140
AuNP	PEG	-SH-Au	Cardiac ventricles and great vessels in rat	CT	142
AuNPs	Poly(ethylene glycol)-b-poly(ε-capralactone)	Non-covalent	HT1080 flank tumors in mice	CT	146
Au nanocluster	Polyacrylic Acid	-COO-Au	Hepatocarcinoma cell line	CT	151
AuNR	polyelectrolyte-dye (poly-d-lysine)	Electrostatic	M1 macrophages	PA	160
AuNSt	DSPE-PEG2000-Folate	Self-assembly	A549 cell in mice	CT/PA	161
AuNR	Reduced graphene oxide	Hydrogen bonding and electrostatic interactions	U87MG tumor in mice	PA	165
AuNP	Glycol-chitosan-coated	-NH_2_-Au	MDA-MB 231 cells	PA/DFCI	166
AuNP	Fucoidan	-OH and -NH- Au	MDA-MB-231 cells	PA	167
AuNR	PEG	-SH-Au	Prostate cancer	PA	6
AuNP	Polystyrene	Gold layer deposited on polystyrene beads	DNA mapping	SERS	173
AuNP	Graphene-PDDA	Electrostatic	Adenine and S. aureus mapping	SERS	174
Au nanocrescent	Polystyrene	Electro deposition	Biomolecule	SERS	178
AuNSt	Polyvinyl alcohol	-OH-Au	MCF and J744 cell ines	SERS	185
AuNR	PEG	-SH-Au	Sentinel lymph node	SERS	188
